# Cross-enhancement of ANGPTL4 transcription by HIF1 alpha and PPAR beta/delta is the result of the conformational proximity of two response elements

**DOI:** 10.1186/gb-2014-15-4-r63

**Published:** 2014-04-10

**Authors:** Tsuyoshi Inoue, Takahide Kohro, Toshiya Tanaka, Yasuharu Kanki, Guoliang Li, Huay-Mei Poh, Imari Mimura, Mika Kobayashi, Akashi Taguchi, Takashi Maejima, Jun-ichi Suehiro, Akira Sugiyama, Kiyomi Kaneki, Hirofumi Aruga, Shoulian Dong, Junko F Stevens, Shogo Yamamoto, Shuichi Tsutsumi, Toshiro Fujita, Xiaoan Ruan, Hiroyuki Aburatani, Masaomi Nangaku, Yijun Ruan, Tatsuhiko Kodama, Youichiro Wada

**Affiliations:** 1 Division of Nephrology and Endocrinology, School of Medicine, The University of Tokyo, 7-3-1, Hongo, Bunkyo-ku, Tokyo 113-8655, Japan; 2 Research Center for Advanced Science and Technology, The University of Tokyo, 4-6-1, Komaba, Meguro-ku, Tokyo 153-8904, Japan; 3 Department of Translational Research for Healthcare and Clinical Science, Graduate School of Medicine, The University of Tokyo, 7-3-1, Hongo, Bunkyo-ku, Tokyo 113-8655, Japan; 4 Jackson Laboratory for Genomic Medicine, 400 Farmington Ave, Farmington, CT 06032, USA; 5 Genome Institute of Singapore, 60 Biopolis Street, #02-01, Genome 138672, Singapore; 6 Tokyo New Drug Research Laboratories, Kowa Company Ltd, 2-17-43, Noguchicho, Higashimurayamashi, Tokyo 189-0022, Japan; 7 Thermo Fisher Scientific, 180 Oyster Point Boulevard, South San Francisco, CA 94080, USA; 8 Radioisotope Center, The University of Tokyo, 2-11-16, Yayoi, Bunkyo-ku, Tokyo 113-0032, Japan

## Abstract

**Background:**

Synergistic transcriptional activation by different stimuli has been reported along with a diverse array of mechanisms, but the full scope of these mechanisms has yet to be elucidated.

**Results:**

We present a detailed investigation of hypoxia-inducible factor (HIF) 1 dependent gene expression in endothelial cells which suggests the importance of crosstalk between the peroxisome proliferator-activated receptor (PPAR) β/δ and HIF signaling axes. A migration assay shows a synergistic interaction between these two stimuli, and we identify angiopoietin-like 4 (*ANGPTL4*) as a common target gene by using a combination of microarray and ChIP-seq analysis. We profile changes of histone marks at enhancers under hypoxia, PPARβ/δ agonist and dual stimulations and these suggest that the spatial proximity of two response elements is the principal cause of the synergistic transcription induction. A newly developed quantitative chromosome conformation capture assay shows the quantitative change of the frequency of proximity of the two response elements.

**Conclusions:**

To the best of our knowledge, this is the first report that two different transcription factors cooperate in transcriptional regulation in a synergistic fashion through conformational change of their common target genes.

## Background

The vascular system sits at the center of oxygen delivery in mammals, and its inner layer endothelial cells play an essential role in network formation. In addition to the physiological angiogenesis that occurs in wound healing and during aerobic exercise, hypoxia is involved in various pathological conditions, for example, cardiovascular disease, diabetic complications, inflammatory diseases and cancer. Poor perfusion of vital organs, including the brain, heart, liver and kidney, can result in hypoxia and critical loss of function. In the core of solid tumors, oxygen demand surpasses the capacity of feeding arteries and the cells are exposed to hypoxia, sometimes with deleterious effects on the progress of the disease. In both contexts, the endothelium is the first cell layer that senses hypoxia as well as changes in hemodynamic forces and blood-borne signals, and this evokes the first step in response to hypoxia, namely angiogenesis [1]. Responding to a demand for more oxygen, endothelial cells migrate and proliferate to form solid endothelial cell sprouts into the stromal space through the induction of a series of gene transcriptional events required for an increased oxygen supply [2].

In the gene regulation that takes place under hypoxia, hypoxia-inducible factor (HIF)1 is regarded as one of the master gene regulators [3] and we previously reported genome-wide analysis of HIF1α location in endothelial cells [4]. Angiogenesis is enhanced by HIF, and it is further orchestrated by various other angiogenic factors, including vascular endothelial growth factor (VEGF) [5], basic fibroblast growth factor (bFGF) [6], angiopoietins and angiopoietin-like (ANGPTL) proteins [7]. In addition to HIF1, another transcription factor (TF), peroxisome proliferator-activated receptors (PPAR)β/δ is reported to participate in angiogenesis [8,9]. PPARs are known to be important in the regulation of numerous biological processes, including lipid metabolism [10], adipocyte differentiation [11,12], cell proliferation [12] and inflammation [13]. To date, three PPAR isotypes have been identified, PPARα, PPARβ/δ and PPARγ. PPARα and PPARγ play a crucial role in lipid metabolism [10], and reports from various groups, including ours, have shown that PPARβ/δ, as well as the other isotypes, also regulate lipid metabolism [14]. In a recent study it was reported that the PPARβ/δ agonist GW501516 stimulated human umbilical vein endothelial cell (HUVECs) proliferation dose-dependently [9], promoted endothelial tube formation, and increased angiogenesis [8]. Another PPARβ/δ agonist, GW0742, or muscle-specific overexpression of PPARβ/δ, also promoted angiogenesis in mouse skeletal muscle [15]. Additional evidence further suggested that PPARβ/δ is one of the important TFs participating in the angiogenic network in endothelial cells [16,17]. These lines of evidence are strongly suggestive of a role for PPARβ/δ in angiogenesis.

Although several key TFs have been shown to be involved in angiogenesis, the detailed underlying hierarchical or mutual interaction of multiple cascades is only partially understood [16]. To dissect the molecular mechanism of crosstalk in angiogenesis, we selected two important angiogenic stimuli, hypoxia and PPARβ/δ agonist stimulation, and investigated the molecular mechanism by which these two signals in concert are able to enhance a common angiogenesis-related target gene.

In this study, we are focusing on the new molecular mechanism where conformational change could contribute to the co-operative transcriptional regulation of a common target by two different TFs. It was previously reported that synergistic transcription could be achieved by different TFs through enhanceosomes [18,19], which are complexes made from proteins binding to regulatory elements of genes. Apart from the enhansceosome concept, which emphasizes the diversity of TF specificity, our findings on synergistic transcription suggest that chromatin structural changes are inseparable from the transcription machinery.

## Results

### Endothelial cell migration is synergistically enhanced by hypoxic and PPARβ/δ agonist stimuli

**Figure 1:**
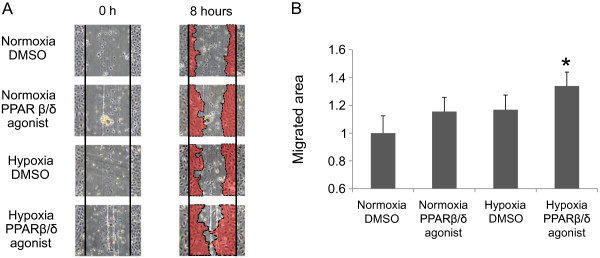
**Endothelial cell migration is synergistically enhanced by hypoxia and the PPARβ/δ agonist. (A)** Images acquired for each stimulus at 0 hour and 8 hours. HUVECs were treated with the PPARβ/δ agonist (GW501516 100 nM) and/or hypoxia (1% O_2_) for 8 hours. The vertical lines indicate the edge of the scratch and the red colored regions at 8 hours show the cell migratory area. Representative images are shown. **(B)** Quantification of the area of endothelial cell migration. The area colored in red (Figure 1A) was measured and the size relative to the untreated condition is indicated. Data (mean ± standard deviation) are representative of three independent experiments with similar results. *P < 0.05 compared with the untreated condition.

To confirm the physiological effect of hypoxia and the PPARβ/δ agonists, and to evaluate the physiological crosstalk of these angiogenic stimuli in endothelial cells, we applied PPARβ/δ and hypoxia to HUVECs and studied the effect on cellular migration function by using a monolayer-wound healing assay. Figure 1A shows the distribution of the cells before and after the stimuli. Quantification of the endothelial cell migratory area (the red area in Figure 1A) is shown in Figure 1B. To avoid the effect of VEGF in the media, the assay was performed using endothelial culture media without any growth factors or fetal bovine serum (FBS). PPARβ/δ and hypoxia individually tended to be associated with greater recovery in HUVECS than normoxia and DMSO, but this was not statistically significant. However, simultaneous application of both stimuli resulted in a significant increase in migration of endothelial cells compared to untreated control. This finding suggested that this experimental motif could be applied to elucidate the synergistic activation that is exerted through PPARβ/δ and HIF1α in endothelial cell function. Therefore, we focused on dissecting the molecular mechanism underlying the synergistic effect of the two stimuli.

### Genome-wide analysis of PPARβ/δ and/or hypoxia-induced genes in endothelial cells identified ANGPTL4 as the common target gene

**Figure 2:**
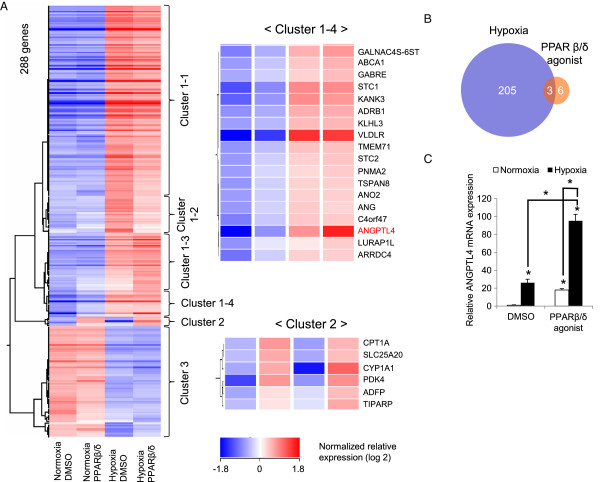
**Genome-wide analysis of PPARβ/δ and/or hypoxia-induced genes in endothelial cells identified *ANGPTL4 *as the common target gene. (A)** Left panel: clustering analysis performed using the selected genes. Right upper panel: genes with their expression profiles placed into the cluster 1-4, which included the genes that responded to both PPARβ/δ agonist and hypoxia. Right lower panel: genes and their expression profiles in cluster 2, which contained the genes induced by the PPARβ/δ agonist, but not hypoxia. **(B)** Venn diagram of the PPARβ/δ agonist and/or hypoxia-induced genes. Based on microarray analysis, after normalization and filtering, gene expression under PPARβ/δ agonist (GW501516 100 nM) or hypoxia (1% O_2_) stimulation (24 hours) in HUVECs was compared with no stimulation (DMSO + normoxia), and then genes that had a fold change ≥1.5 were selected. **(C)** Quantitative RT-PCR analysis of *ANGPTL4* for both types of stimulation. HUVECs were stimulated with the PPARβ/δ agonist (GW501516 100 nM) and/or hypoxia (1% O_2_) for 24 hours. Data (mean ± standard deviation) are representative of two independent experiments with similar results. *P < 0.001 compared between the indicated conditions.

To estimate the possible interaction of the PPARβ/δ and HIF1α signaling pathways in a more comprehensive manner, we performed transcriptome analysis using microarrays after 24 hours of treatment with a PPARβ/δ-selective agonist (GW501516, 100 nM) and/or hypoxic (1% O_2_) stimulation. After normalization and filtering, gene expression change against the normoxia-DMSO sample was calculated. A scatter plot of normalized intensity values of all genes under two conditions is shown in Figure S1A,B in Additional file 1. The PPARβ/δ agonist-induced genes (Figure S1A in Additional file 1) were compared with the hypoxia-induced genes (Figure S1B in Additional file 1). In general, the number of genes induced by hypoxia was much larger than that induced by the PPARβ/δ agonist. To extract the genes responsive to either of the stimuli, genes that had a fold change ≥2.0 were selected; 288 genes remained. Hierarchical cluster analysis was performed on these, and the genes were classified into three clusters (Figure 2A; Figure S2 in Additional file 1). Genes that exhibited induction under hypoxia are in cluster 1 (Figure 2A; Figure S2 in Additional file 1). This cluster was then subclustered into four conditions (cluster 1-1 to 1-4), and the genes that also exhibited up-regulation by the PPARβ/δ agonist were classified into cluster 1-4 (Figure S2 in Additional file 1). Likewise, the genes induced by the PPARβ/δ agonist, but not hypoxia, were placed into cluster 2 (Figure 2A; Figure S2 in Additional file 1). As illustrated by the Venn diagram in Figure 2B, 208 out of 288 genes exhibited induction by hypoxia, and 9 genes were induced by GW501516, with an overlap of 3 genes (fold change ≥1.5). As shown in Table S1 in Additional file 2, the overlapped genes were angiopoietin-like 4 (*ANGPTL4*), arrestin domain-containing 4 (*ARRDC4*) and leucine rich adaptor protein 1-like (*LURAP1L*), exhibiting 35.3-, 2.5-, 2.2-fold induction under PPARβ/δ agonist treatment and hypoxia compared to no stimulation, respectively (Table S1 in Additional file 2). All of these three genes were placed in cluster 1-4 (Figure 2A, right upper panel).

Genes (fold change ≥1.5, shown in the Venn diagram in Figure 2B) up-regulated by the PPARβ/δ agonist are listed in Table S1 in Additional file 2, and the gene most highly induced by the PPARβ/δ agonist was also *ANGPTL4*, which displayed a 7.0-fold induction compared with vehicle treatment. The genes induced by the PPARβ/δ agonist but not hypoxia were included in cluster 2 (Figure 2A, the lower right panel). In addition, *ANGPTL4* was the gene most highly induced by hypoxia, having a 20.1-fold induction compared to normoxia (Tables S1 and S2 in Additional file 2). A scatter plot of the fold change values induced by the PPARβ/δ agonist or hypoxia compared with no treatment is shown in Figure S3 in Additional file 1, again showing that no other genes responded to the same level as *ANGPTL4* under the treatments. Up-regulation of *ANGPTL4* by PPARβ/δ agonist treatment and hypoxic stimulation were confirmed by quantitative RT-PCR (qRT-PCR), with the result showing synergistic activation (Figure 2C). *ANGPLT4* transcription was measured by amplifying nascent transcript with primers recognizing intron-exon junctions (Figure S4 in Additional file 1) and similar results were obtained (the primers for qRT-PCR are shown in Table S3A in Additional file 2). Western blotting indicated that ANGPTL4 was also synergistically generated by the dual stimulation (Figure S5 in Additional file 1). Increased amounts of recombinant ANGPTL4 protein were confirmed to enhance migration of endothelial cells (Figure S6 in Additional file 1). Taking these data into account, we focused on *ANGPTL4* as a key motif in the elucidation of the molecular crosstalk mechanism.

### Whole genome analysis of PPARβ/δ and HIF1α binding sites in endothelium confirmed ANGPTL4 as the common target gene

To extract the genes that are directly regulated by PPARβ/δ, we carried out chromatin immunoprecipitation (ChIP) using a PPARβ/δ antibody in HUVECs treated with PPARβ/δ agonist stimulation for 24 hours, followed by deep sequencing (ChIP-seq). In total 38,936,258 reads were aligned and 77.6% of the total reads were aligned uniquely to the non-repeating human genomic sequence. Next, we calculated the enrichment of the PPARβ/δ ChIP DNA fragments compared with the input, and determined the significant PPARβ/δ binding sites according to the QuEST algorithm [20]. In total, 364 binding regions were identified as PPARβ/δ enrichment sites under PPARβ/δ agonist treatment (Figure S7A in Additional file 1). To investigate the correlation of the PPARβ/δ binding regions and the nearest known transcripts, we divided the regions into five sections based on the distance from the transcription start site (TSS) of the corresponding genes. As shown in Figure S7A in Additional file 1, 82% were positioned in intergenic regions under PPARβ/δ agonist treatment, 6% were located upstream (25 kbp to 1 kbp upstream of the TSS), and 3% were located on the TSS (within a range of 1 kbp upstream of the TSS to the first intron).

**Figure 3:**
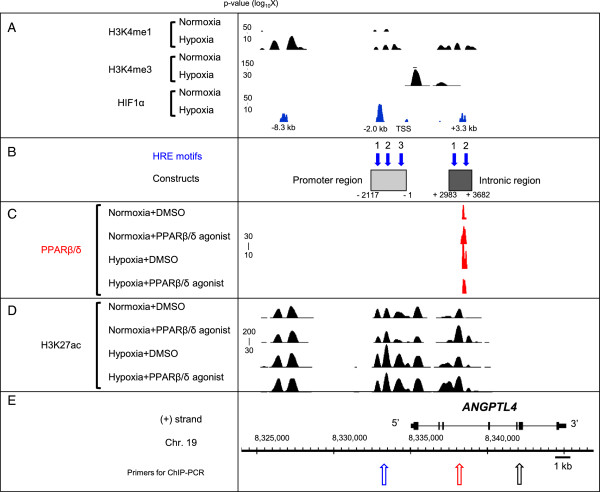
**ChIP-seq analysis (H3K4me1, H3K4me3, HIF1α, PPARβ/δ, H3K27ac) of the *ANGPTL4 *gene and construct design for the reporter assay.** The displayed enrichment scores for ChIP-seq were calculated by QuEST and visualized with IGB software (Affymetrix). **(A)** ChIP-seq data for H3K4me1, H3K4me3 and HIF1α under normoxia or hypoxia. HUVECs were stimulated with normoxia or hypoxia (1% O_2_) for 24 hours. The kilobase values show the distance from the TSS of *ANGPTL4*. **(B)** Constructs shown in Figure 4 are shown in the frame with their names. Both the constructs were cloned into a pGL3 vector. The displayed hypoxia response element (HRE) motifs (blue arrows and numbers) were estimated by MatInspector (Genomatix). The numbers under the constructs show the distance from the TSS of *ANGPTL4* (in base pairs). **(C,D)** ChIP-seq data for PPARβ/δ **(C)** and H3K27ac **(D)** using HUVECs stimulated with the PPARβ/δ agonist (GW501516 100 nM) and/or hypoxia (1% O_2_) for 24 hours. **(E)** Basic information for the analysis. The numbers indicate the location on chromosome 19 using the hg18 build. The arrows show the location of primers for ChIP-PCR in Figure 4.

To validate the ChIP-seq data for PPARβ/δ, we performed a motif search of PPARβ/δ binding sites (Figure S7A in Additional file 1, right panel), and the generated sequences perfectly matched the known PPAR binding motifs. The qRT-PCR and ChIP-seq data for representative genes responsive to PPARβ/δ or hypoxia are shown in Figure S7B,C in Additional file 1. Pyruvate dehydrogenase kinase, isozyme 4 (*PDK4*), carnitine palmitoyltransferase 1A (*CPT1A*) and solute carrier family 25, member 20 (*SLC25A20*), all listed in Table S1 in Additional file 2, are well-known PPARβ/δ target genes. In terms of the hypoxia-induced genes, the genes expressed more than two-fold under hypoxia compared with normoxia are shown in Table S2 in Additional file 2. The qRT-PCR and ChIP-Seq data for three representative genes are shown in Figure S7B,C in Additional file 1. Vascular endothelial growth factor (*VEGFA*), solute carrier family 2, member 1 (*SLC2A1*) and adenylosuccinate synthase like 1 (*ADSSL1*) are hypoxia induced genes, showing 3.3-, 6.5- and 4.3-fold induction compared with normoxia, respectively (Table S2 in Additional file 2). Using previously obtained data on HIF1α binding sites, the commonly bound genes were extracted, and the binding of PPARβ/δ and HIF1α at the *ANGPTL4* locus was confirmed (Figure 3A,C). ChIP-seq analysis revealed that *ANGPTL4* is the only gene bound by PPARβ/δ and HIF1α among the common target genes identified by microarray analysis. In addition, the gene most highly induced by the two different regulators was *ANGPTL4*.

### ANGPTL4 is regulated by both PPARβ/δ and HIF1α signaling cascades

To determine whether *ANGPTL4* was induced through PPARβ/δ and HIF1α signaling, we performed a series of experiments using small interfering RNA (siRNA) against PPARβ/δ and/or HIF1α. As shown in Figure S8A,B in Additional file 1, the efficiency of the siRNA-mediated knockdown of PPARβ/δ was over 80% at the mRNA level with or without its agonist. The efficiency of the knockdown of HIF1α was over 90% (Figure S8C,D in Additional file 1). Almost complete inhibition of *ANGPTL4* induction by the agonists was achieved by the siRNA against PPARβ/δ (Figure S9A in Additional file 1). *ANGPTL4* mRNA levels were reduced by over 70% under hypoxia using either of the two siRNAs against HIF1α (Figure S9B in Additional file 1). When both stimuli were applied, *ANGPTL4* mRNA levels were reduced by over 60% using a combination of si-PPARβ/δ oligo1 and si-HIF1α oligo 1 (Figure S9C in Additional file 1). These data confirmed that the up-regulation of *ANGPTL4* by the PPPAβ/δ agonist and/or hypoxia is dependent on both PPARβ/δ and HIF1α.

### Identification of the functional hypoxia response element of ANGPTL4

Since *ANGPTL4* is downstream of the two transcription cascades and is commonly activated, we tried to dissect the molecular mechanism of the dual enhancement. We therefore set out to identify a functional hypoxia response element (HRE) in *ANGPTL4*. HUVECs were stimulated with hypoxia (1% O_2_) and incubated for 24 hours, followed by identification of histone modifications (monomethylated histone 3 lysine 4 (H3K4me1), trimethylated histone 3 lysine 4 H3K4me3, and acetylated histone 3 lysine 27 (H3K27ac)) and HIF1α binding sites by ChIP-seq. As shown in Figure 3A, no binding of HIF1α was observed under normoxia in the *ANGPTL4* gene locus, but five HIF1α binding sites appeared under hypoxia. H3K4me3, known as a promoter marker [21], was only observed for *ANGPTL4* under hypoxia, suggesting that the *ANGPTL4* locus became activated under hypoxia. Three out of the five potential HIF1α binding sites were also accompanied by two markers of enhancer activity, H3K4me1 [22] and H3K27ac [23-25]. As the resolution of ChIP-seq is about 100 bp [26], to narrow the binding locus, HRE motifs were predicted in both the promoter and intron regions (blue arrows in Figure 3B) using MatInspector software (Genomatix). Based on co-localization of HIF1α binding, histone marks, and HRE motifs, we named these enhancer/promoter regions the 'promoter region' and the 'intronic region', from 5′ upstream, respectively (Figure 3B). Considering this classification, we made a series of constructs for a subsequent reporter assay. From the 5′ end, we named the HRE motifs 'Pro-HRE1' to 'Pro-HRE3', and 'Int-HRE1' and 'Int-HRE2' (Figure 3B).

**Figure 4:**
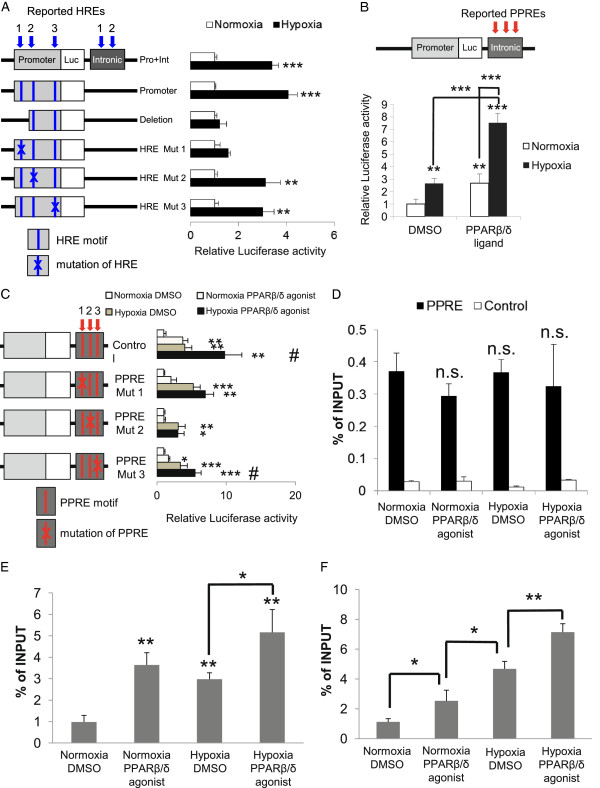
**Identification of the functional HRE and PPAR-response element on *ANGPTL4, *the quantity of PPARβ/δ binding to the third intron of *ANGPTL4 *and histone acetylation level of the response elements. (A)** Reporter assay with HRE mutations in intronic regions in HUVECs. HUVECs were transfected and stimulated as shown in Material and methods. The reported HREs in the promoter and intronic region located in constructs and shown by the blue arrows with numbers. **(B)** HUVECs were transfected with the construct containing known PPAR-response elements (PPREs; red arrows in upper panel) and were stimulated with the PPARβ/δ agonist GW501516 (100 nM) and/or hypoxia (1% O_2_) for 24 hours. Comparison was done between the indicated conditions. **(C)** Reporter assay for PPRE mutations. Cells were transfected with each construct and stimulated with the PPARβ/δ agonist and/or hypoxia for 24 hours (as above). The reported PPREs are shown by the red arrows with numbers. Comparison was with the untreated condition, and ^#^*P* < 0.05 compared to the hypoxic condition. **(D)** ChIP-PCR of PPARβ/δ under the four conditions. Location of primers is shown in Figure 3C. **(E)** ChIP-PCR of H3K27ac under the four conditions around the PPARβ/δ binding site. Primers were designed for an upstream region close to the PPARβ/δ binding site at the third intron of *ANGPTL4* (Figure 3E). **(F)** ChIP-PCR of H3K27ac under the four conditions around the HIF1α binding site. Primers were designed for a downstream region close to the HIF1α binding site 2 kbp upstream of the TSS (blue open arrow in Figure 3E). Primers for ChIP-PCR are listed in Table S5 in Additional file 2. Comparison was with normoxia-DMSO sample or between indicated pairs. Data (mean ± standard deviation) are representative of three **(A to C)**, or two **(D and F)** independent experiments with similar results. ***P < 0.001, **P < 0.01, *P < 0.05; n.s., not significant.

After luciferase activity was up-regulated in the presence of the promoter region under hypoxia (data not shown), we made a further series of deletion mutant constructs and HRE motif-mutated constructs to identify the hypoxia responsive sites (Table S3B in Additional file 2). Using these mutated constructs and the promoter and intronic regions, a luciferase assay was performed in HUVECs under hypoxia (Figure 4A). The luciferase activity of the promoter and intronic regions (shown in the first line) was unchanged compared to the promoter construct (shown in the second line) (Figure 4A). Thus, the intronic region did not play an essential role under hypoxia. Among the six constructs, the luciferase activity under hypoxia was not significantly different except for two constructs; the Pro-HRE1 deletion construct and the HRE mutation 1 construct. However, the HRE mutation 2 construct and HRE mutation 3 construct were up-regulated to some extent. Thus, Pro-HRE1 in the promoter region, which is located 2.0 kb upstream of the TSS, was important for hypoxia responsive induction.

### Synergistic activation of ANGPTL4 transcription by hypoxia and PPARβ/δ agonist in HUVECs and identification of the functional PPAR-response element

As shown in Figure 2C, the induction of *ANGPTL4* with both stimuli was approximately five times higher than that of hypoxia or the PPARβ/δ agonists alone, suggesting that there is a synergistic activation mechanism between the two stimuli in HUVECs. Based on the finding that the PPARβ/δ binding site was identified by ChIP-seq at the third intron of *ANGPTL4* (Figure 3C), where functional HIF1α binding sites had already been identified (Pro-HRE1 in Figure 3B), a reporter assay was carried out using constructs containing the two essential units, the promoter region, which is important for HIF1α stimulation, and the intronic region, which is utilized for PPARβ/δ signaling. As shown in Figure 4B, the activity of this construct was greater than up-regulation from a single stimulus. Thus, we considered the constructs shown in Figure 4B to contain the units essential for synergistic activation, and we made each of the mutated PPAR-response element (PPRE) constructs shown in Figure 4C to identify the functional PPRE. Reporter activity induction was compared to a non-mutated control construct (Figure 4C, top), under four different conditions in three kinds of mutation construct. Among them, the induction activity of the PPRE mutation 2 construct was suppressed significantly, and the induction by PPARβ/δ agonist under normoxia was canceled. Even under hypoxia, the effect of the PPAR agonist was diminished in this construct. These results suggested that all of the three PPREs contributed to some extent to the transcriptional enhancement, but PPRE 2 at the third intron had the most profound effect on the regulation of *ANGPTL4* through PPARβ/δ agonist stimulation.

### Stimuli induce the histone acetylation level of the response elements and not the quantity of PPARβ/δ binding to the third intron of ANGPTL4

As in the case of HIF1α recruitment, we originally hypothesized that PPARβ/δ binding might be increased in the course of the synergistic activation. Thus, we analyzed PPARβ/δ binding by ChIP-seq under four conditions: no stimulation (DMSO + normoxia), PPARβ/δ agonist stimulation (GW501516 + normoxia), hypoxia (1% O_2_ + DMSO), and both PPARβ/δ agonist and hypoxia (GW501516 + 1% O_2_) for 24 hours. Unexpectedly, the locations and distribution patterns of the PPARβ/δ binding (shown in red) to *ANGPTL4* did not change under the four conditions (Figure 3C). Furthermore, the quantity of PPARβ/δ binding to *ANGPTL4* was compared by ChIP-PCR using primers of the PPARβ/δ binding site at the third intron of *ANGPTL4* (Figure 3C; the primers for ChIP-PCR are listed in Table S3C in Additional file 2), and the level of PPARβ/δ binding under the four conditions was equivalent (Figure 4D).

Thus, we speculated that PPARβ/δ might be activated without any distribution change, and to test this notion, we determined whether the activity of the enhancer was affected. Previously, CBP/p300-mediated H3K27 acetylation in PPARβ/δ-dependent transcription was reported [25], so we evaluated the intensity of H3K27ac, a marker of enhancer activity. First, ChIP-seq analysis using an anti-H3K27ac antibody was performed under the four conditions and its distribution patterns on a genome-wide scale were analyzed. In general, the number of acetylation sites was increased by any of the forms of stimulation by 12 to 13%, but its whole genome distribution patterns did not change significantly under the different conditions (Figure S10 in Additional file 1). Approximately 70% of the H3K27ac was found at the genes under all the conditions. In detail, 4% of H3K27ac was located in an upstream region, 7% at the TSS, 6 to 7% in the 5′ UTR, 15 to 16% in the first intron, 23 to 24% in other introns, 5% in exons, 1% in the 3′ UTR and 4 to 5% in downstream regions.

In terms of *ANGPTL4*, consistent with the general tendency, the binding distribution of H3K27ac in *ANGPTL4* did not change depending on the conditions (Figure 3D), but the intensity of H3K27 acetylation did change with the different types of stimulation. To compare this quantitatively, we performed ChIP-PCR using the primers designed for the HRE and PPRE sites (the primers for ChIP-PCR are listed in Table S3C in Additional file 2). The level of H3K27 acetylation around the functional PPRE (the site indicated with the open red arrow in Figure 3E) was 3.7 times more enhanced by the PPARβ/δ agonist, which is consistent with the ChIP-seq data (Figure 4E). Surprisingly, however, even with hypoxic stimulation, the acetylation level around PPRE was 3.0-fold up-regulated, and 5.3-fold induction was observed with a combination of hypoxia and PPAR agonist (Figure 4E). The same phenomenon was observed around the functional HRE (Figure 4F). The level of H3K27 acetylation around the HRE (the site indicated with the open blue arrow in Figure 3E) was 4.2 times more enhanced under hypoxia. In addition, the acetylation around HRE was 2.3 times increased even with the PPARβ/δ agonist alone, and 6.4 times with the combination of stimuli (Figure 4F). These results suggest that hypoxia and PPARβ/δ together cross-enhance the intensity of the TF-bound enhancer sites.

### HIF1α and PPARβ/δ change the chromatin conformation in the ANGPTL4 locus

**Figure 5:**
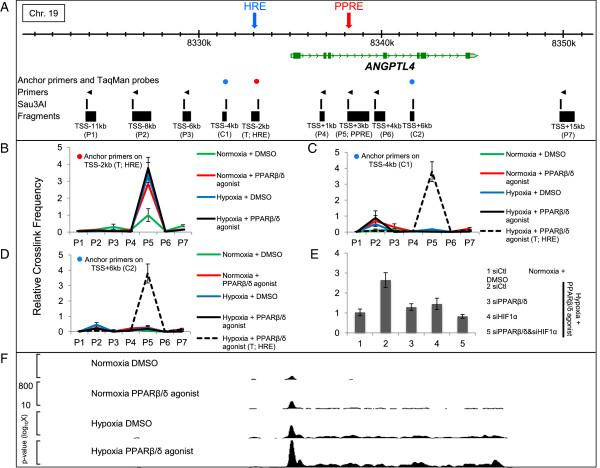
**Chromatin conformation was changed at the *ANGPTL4 *locus by HIF1α and PPARβ/δ. (A)** Schematic diagram of the *ANGPTL4* gene locus and 3C experimental settings. The colored circles show the position of anchor primers and TaqMan probes (red for target anchor, blue for control anchor). Arrowheads show the positions of primers. The digestion sites of *Sau3AI* are shown with the generated fragments below. The distance from the TSS of *ANGPTL4* is shown at the bottom with the primer name. The functional HRE and PPRE are shown with blue and red arrows. The genomic location on the chromosome based on the hg18 build is shown at the top. **(B)** 3C assays based on HRE fragment. Anchor primer and TaqMan probe were designed on HRE fragment and the other primers were designed for analyzing the crosslinking frequency of the HRE and the other fragments (Figure 5A). HUVECs were treated with four conditions, then the relative frequencies were compared among them. **(C,D)** 3C assays based on the control 1 **(C)** and control 2 **(D)** fragments. Anchor primer and TaqMan probe were designed on the fragment located 4 kb upstream of the TSS (C1), and on the fragment located 6 kbp downstream of the TSS (C2). The other primers are the same as in **(B)**. To compare the data properly, the relative frequency data under dual stimulations in **(B)** are shown by the dotted line. Primers for 3C experiments **(B-D)** are shown in Table S3D in Additional file 2 and the locations of primers are shown in Figure 5A. **(E)** 3C assay of the target motif (HRE and PPRE) using siRNA against HIF1α and/or PPARβ/δ. HUVECs were transfected with siRNAs and, 48 h later, stimulated for 24 hours. **(F)** ChIP-seq analysis (RNA polymerase II) of *ANGPTL4*. Data (mean ± standard error of the mean) are representative of three independent experiments with similar results **(B-E)**.

To dissect the molecular mechanism by which the two different signaling cascades communicate with each other, and with the intention of providing a physical basis for the phenomenon, we considered the possibility that a change in chromatin conformation might participate in the cross-talk, since the main role of the enhancer is to form a chromatin loop through spatial proximity with the TSS [4,27]. To evaluate the proximity frequency of the two response sites (HRE and PPRE) under the four different conditions, quantitative chromatin conformation capture (3C) assay was performed. As shown in Figure 5A, the functional HRE (blue arrow) and PPRE (red arrow) are separated by approximately 5.3 kb, and to perform the 3C assay, we chose *Sau3AI*, a four base pair cutter, for DNA fragmentation. The primers and TaqMan probes for the 3C target analysis were designed using both of the fragments containing the functional HRE or PPRE (Figure 5A; and Table S3D in Additional file 2). Figure 5B shows the results of the TaqMan-3C assay, including the target locus shown with the red circle in Figure 5A. Except for the target region, no increased interaction was observed. In the case of either stimulation, compared with the control condition of normoxia and DMSO (green versus red or blue lines in Figure 5B), the frequency of crosslinking between the HRE and PPRE was increased. The same crosslinking frequency was observed for the combination of hypoxia and PPARβ/δ agonist (Figure 5B, black line). These results suggest that both of the single stimulations brought one responsive site into proximity of the other responsive site. To validate the reliability of the 3C assay, PCR products were directly sequenced and the conjunction of the HRE and PPRE fragments mediated by the restriction site was confirmed (Figure S11 in Additional file 1). To see the specificity of the looping between HRE and PPRE, we also performed a TaqMan-3C assay with two different control anchor primers (blue circles in Figure 5A). Figure 5C shows the results with the anchor primer at the fragment located -4 kb from the TSS of *ANGPTL4*, and Figure 5D shows these using the primers 6 kb downstream of the TSS of *ANGPTL4*. In Figure 5C, the relative crosslinking frequencies between P2 (TSS-8 kb) and control anchor region 1 (C1; TSS-4 kb) were slightly increased under the stimulations, but no looping was observed between C1 and the PPRE (P5). Similarly, no increased relative crosslinking frequency was observed between control anchor region 2 (C2) and the PPRE (P5), as expected. These results support that the looping between the HRE and PPRE specifically occurs upon stimulation.

To determine whether HIF1α or PPARβ/δ binding requires the observed chromatin conformational change at the *ANGPTL4* locus, we treated cells with siRNA against HIF1α and/or PPARβ/δ and performed quantitative 3C assays under stimulation with both hypoxia and the PPARβ/δ agonist (Figure 5E). Though the effect of hypoxia plus the PPAR agonist was measurable (1 versus 2 in Figure 5E), the frequency was changed by a reduction of either PPARβ/δ (2 versus 3 in Figure 5E) or HIF1α (2 versus 4 in Figure 5E) or of a combination of both factors (2 versus 5 in Figure 5E), supporting the notion that HIF1α and/or PPARβ/δ are involved in chromatin loop formation at the HRE and PPRE of the *ANGPTL4* locus.

### Additive effect on active RNA polymerase II recruitment *by HIF1α and PPARβ/δ*

The 3C data (Figure 5B-E) strongly suggest that a higher frequency of spatial proximity of two response elements is beneficial to synergistic induction of *ANGPTL4* by hypoxia and the PPARβ/δ agonist in HUVECs (Figure 2C). The luciferase activity result for the *ANGPTL4* construct (Figure 4B) also supports this notion. To confirm the conformation change is associated with more efficient recruitment of active RNA polymerase II (Pol II), ChIP-seq analysis using anti-phospho C-terminal domain (CTD) of Pol II under the four conditions was performed. As shown in Figure 5F, more Pol II was recruited to the TSS of *ANGPTL4* with either hypoxic (second line in Figure 5F) or PPARβ/δ agonist stimulation (third line in Figure 5F). Furthermore, the highest degree of Pol II distribution in *ANGPTL4* was observed under the dual stimulation (fourth line in Figure 5F) and this phenomenon was confirmed by ChIP-PCR of Pol II (Figure S12 in Additional file 1). These findings confirm that the conformational change caused by the dual stimulation resulted in additive localization of Pol II in the target motif.

## Discussion

The angiopoietin/angiopoietin-like gene family encodes a glycosylated, secreted protein with a fibrinogen carboxy-terminal domain. In vascular cells, the angiopoietins act as major regulators of angiogenesis and vascular permeability through binding to the Tie-2 receptor [7]. Angiopoietin-like proteins (ANGPTL1 to 7) share structural and functional properties with angiopoietins, but do not bind to the Tie-2 receptor [28]. This study shows that, under hypoxia and PPARβ/δ stimulation, *ANGPTL4* was intensely expressed (Figure 2C; Table S1 in Additional file 2). These results confirm the previous finding that *ANGPTL4* is highly induced by hypoxia in endothelial cells [29,30]. Full length ANGPTL4 exists as an oligomeric complex, and it can be cleaved *in vivo* to release the two domains, resulting in the presence of both the full-length and cleaved forms in plasma. The amino-terminal coiled-coil domain inhibits lipoprotein lipase (LPL) activity and increases plasma triglycerides [31], while the carboxy-terminal FBG-like domain (cANGPTL4) is associated with the integrity of endothelial cells [32]. Since migration of HUVECs was synergistically enhanced by the two stimuli (Figure 1), which induced ANGPTL4, our findings imply that increased carboxy-terminal domain activity might participate by disrupting endothelial junctions by an effect on integrins, as was reported previously [33]. In contrast, however, ANGPTL4-deficient mice displayed decreased vascular integrity in the retina [34] and heart [35]. Furthermore, administration of recombinant ANGPTL4 was effective as a treatment for acute myocardial infarction because it counteracts the increase in permeability observed in re-perfused acute myocardial infarction [35]. Although leukocyte immunoglobulin-like receptor B2 (LILRB2) was identified as an agonist for other members of the ANGPTL family, the agonist for ANGPTL4 is still unidentified [36]. Furthermore, the PPARβ/δ-ANGPTL4 pathway was shown to be involved in tumor cell invasion [37]. Therefore, the role of ANGPTL4 in the inflammatory response *in vivo* needs to be interpreted, taking into consideration communication with other cell types involved in angiogenesis or wound healing.

One of the transcriptional regulators of *ANGPLT4* is HIF1α, and this TF is known to be recruited to target genes immediately after hypoxic stimulation [4]. Another transcription regulator of *ANGPTL4* is PPARβ/δ, which belongs to the nuclear receptor superfamily as a subclass of TFs. In general, gene induction by nuclear receptors only happens when a ligand (agonist) is present. PPAR ligands (agonists) change the PPARs’ structures, resulting in modulation of receptor properties [38]. However, an agonist-independent high basal activity of PPARs has also been reported [39]. In addition, DNA binding of the thyroid receptor, which belongs to the nuclear receptor family (type II nuclear receptor), was reported to not be agonist-dependent [40]. Thus, the recruitment of nuclear receptors is not strictly essential to agonist-dependent gene induction. PPARs are also type II nuclear receptors, and fatty acids, triglycerides, prostacyclin, and retinoic acid are known as endogenous agonists for PPARβ/δ [41]. In our results, the quantity of PPARβ/δ binding at the *ANGPTL4* locus was not changed under the four conditions we tested (Figure 4D), confirming the notion that PPARβ/δ binds to DNA irrespective of the presence of its exogenous agonists. In the absence of agonists, nuclear receptors function by utilizing co-repressors, which maintain the nuclear receptors in a repressed state, and these co-repressors are dismissed when the co-activators are recruited following agonist stimulation [25,42]. Co-activators have been reported in the case of certain TFs, including HIF1α [43], and the involvement of CBP/p300 was linked to histone acetylation [44]. As reported previously, PPARβ/δ-dependent transcription was also linked to CBP/p300-mediated H3K27ac [25]. Taken together, the generation of H3K27ac is a common histone modification shared by the PPARβ/δ and HIF1α signaling cascades, and analyses of the H3K27ac distribution profile allowed us to trace the activity of these two transcriptional regulators.

We compared the ChIP-seq data for HIF1α and PPARβ/δ with those for H3K4me3 as a marker of promoter activity [21], and H3K4me1 [22] in addition to H3K27ac [23-25] as markers of enhancer activity (Figure 3A,D). The potentially active enhancers based upon the co-localization of both TFs and histone modifications were validated by luciferase assays (Figure 4). The PPAR binding region observable near *ANGPTL4* by ChIP-seq in HUVECs was consistent with those reported previously in myofibroblasts [42]. The function of the binding region as an enhancer was supported by reporter assay in endothelial cells, as was reported previously in adipocytes [45] and myofibroblasts [46]. To confirm this, we performed more assays using PPRE deletion constructs. The intensity of H3K27ac at the functional PPRE was enhanced by PPAR agonist addition, but also cross-enhanced by hypoxia (Figure 4E). H3K27ac at the HRE was increased not only by hypoxia, but also by the PPAR agonist (Figure 4F). These findings led us to the notion that the two elements might be located in spatial proximity of one another. Since H3K27ac is acetylated mainly by CBP/p300 [25], histone acetyltransferases might be recruited to both the HRE and PPRE following each stimulus. However, there is no known mechanism to bring CBP/p300 to PPREs or *vice versa* after hypoxia. One explanation for this may be co-instantaneous chromatin conformational change, which could bring about a closer spatial proximity of the HRE and PPRE, and this was indeed shown to be the case by TaqMan-3C assay (Figure 5B-E). As reported previously, this can result in loop formation between the two elements [47]. Therefore, our data suggest the existence of complexes composed of TFs and histone acetyltransferases.

**Figure 6:**
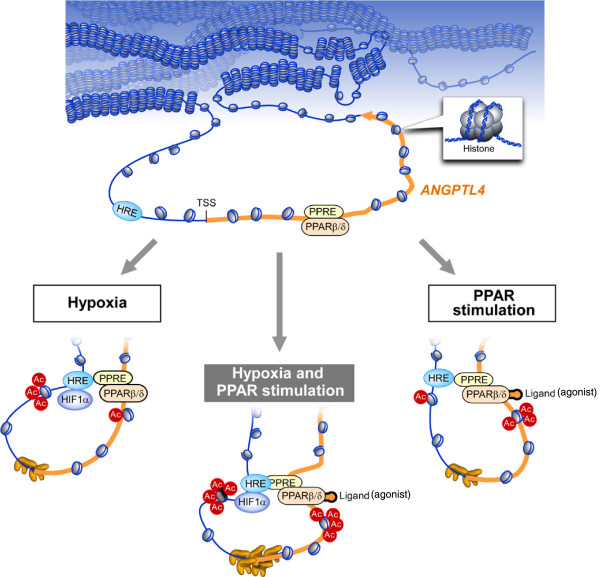
**Schematic diagram of synergistic transcription induction mediated by spatial proximity.** Upper part shows the *ANGPTL4* locus, including the HRE, TSS, and PPRE. PPARβ/δ sits at the PPRE before stimulation. Left: under hypoxia, acetyltransferases approach the HRE locus by virtue of HIF1α binding to the HRE. *ANGPTL4* assumes a loop formation in order to bring the HRE closer to the PPRE. Although the PPRE is not a direct target of HIF1α, this conformation causes acetylation of the PPRE. Right: under PPAR ligand (agonist) stimulation, PPARβ/δ's location does not change but it is chemically modified to the active form. Presumably by virtue of a cofactor of PPAR, the PPRE comes closer to the HRE, resulting in acetylation of the HRE. Center: under dual stimulation, the HRE and PPRE move closer to each other, causing additive acetylation of both regions, and increased amounts of Pol II might be loaded at the TSS, possibly resulting in synergistic transcription activation and crosstalk.

In yeast [48] and mammalian cells [49,50], a mediator complex connects enhancers and core promoters through chromatin conformational changes in combination with cohesin. Further conceptualization of the complexity of transcription initiation postulates the existence of 'transcription factories' [51-53] containing a variety of components, including active Pol II, histone variants, and histone modifiers. As was shown in the context of inflammatory stimulation, specialized factories are supposed to contain appropriate TFs, in this case NF-κB [45]. Likewise, specialized factories for hypoxia might contain HIF1α and for the PPAR agonist might contain PPARβ/δ, which connects factories and enhancers. Therefore, it is plausible that the HIF1α-driven factory would come into contact with the HRE in the 5′ upstream region of the TSS and then change chromatin conformation to bring the HRE closer to the transcribed region of *ANGPTL4* (Figure 5B)*.* This would result in spatial proximity of CBP/p300 and the PPRE, causing histone acetylation of the PPRE (Figure 4E). In contrast, acetylation of the HRE upon PPARβ/δ agonist stimulation (Figure 4F) is difficult to explain unless the transcription complex recruited to the PPRE changes the chromatin conformation such that the HRE and PPRE come into closer proximity. As shown in Figure 5B, this spatial proximity between the HRE and PPRE was observed in the case of each stimulus individually or both stimuli together using the 3C method. Considering the cross-acetylation of histone 3 lysine 27 (Figure 4E,F), this chromatin conformation change might suggest a specialized transcription factory of HIF1α and agonist bound PPARβ/δ. Also, this conformational change might enable one enhancer to be activated by another stimulus, and this may be the mechanism responsible for synergistic activation of common target genes by two kinds of transcription regulators (Figure 6). Knocking down one factor (either PPARβ/δ or HIF1α) under the dual stimulation condition resulted in greatly reduced interaction between the HRE and PPRE, even though the other factor is still intact (Figure 5E). It could be speculated that a specialized factory without a specific TF not only loses its function but also perturbs other native transcription complexes, presumably by taking over Pol II or by blocking appropriate recruitment of another specialized factory, and this model should be tested in the near future. Although the details of the components and dynamics of PPAR-dependent factories needs to be elucidated, these findings might support the existence of specialized transcription factories, as predicted.

To the best of our knowledge, this is the first report of two different TFs cooperating in transcriptional regulation through the conformational change of the target gene. Nuclear receptors have the potential to crosstalk with various other sequence-specific DNA-binding TFs at adjacent sites, resulting in a modification of gene expression. The existence of crosstalk between different TFs has already been reported, and some of the mechanisms have been elucidated [54]. 'Pioneer TFs' such as forkhead box A1 (FoxA1) and estrogen receptor alpha (ERα) provide an example of the interaction mechanisms [55]. The synergistic regulation of *ANGPTL4* by transforming growth factor (TGF)β and PPARβ/δ was reported using a human prostatic stromal myofibroblast cell line (WPMY-1), and looping between the TGFβ response element enhancer and PPAR-response element enhancer was in fact postulated previously, although it was not confirmed [46]. The development of 3C technology enabled us to demonstrate the conformational proximity of the two response elements [26] under different conditions. Our findings imply that a chromatin conformational change may underlie the synergistic gene activation that takes place with different stimuli.

## Conclusions

Chromatin conformation capture and ChIP studies clearly identified that the mechanism of synergistic *ANGPTL4* activation comprises DNA looping and histone modification. The mechanism of synergistic *ANGPTL4* activation provides an important clue to how different types of stimulation interact with each other.

## Materials and methods

### Cell culture

Cells were maintained at 37°C in a humidified 5% CO_2_ incubator. Primary culture of HUVECs was prepared and maintained in EGM-2 MV (Lonza, Basel, Switzerland) containing 5% FBS. Experiments under hypoxic conditions (1% O_2_) were performed in a hypoxic cultivation incubator (Juji Field Co. Ltd, Tokyo, Japan). The cells used in the experiments were from passage 6 or less.

### Chemicals and antibodies

GW501516 (GlaxoSmithKline, London, UK) was used as a PPARβ/δ agonist at a concentration of 100 nM. An anti-PPARβ/δ monoclonal antibody was produced in-house [56]. Mouse monoclonal IgG-Y9705 against human PPARβ/δ was raised in our laboratory by immunizing mice with recombinant baculovirus displaying gp64-fusion proteins containing amino acids 2 to 41 of human PPARβ/δ. The anti-HIF1α (NB 100-134; Novus Biologicals, Littleton, CO, USA), anti-H3K4me3 (ab8580; Abcam, Cambridge, UK) antibodies and anti-ANGPTL4 (ab47046; Abcam) were purchased. The anti-H3K27ac, anti-H3K4me1 [57] and anti-panphospho Pol II antibodies were provided by Dr Kimura of Osaka University (Maejima *et al*., PLoS One *in press*). Recombinant ANGPTL4 (P01) was from Abnova (Taipei, Taiwan).

### Chromatin immunoprecipitation

Two million HUVECs were plated on a 15-cm culture plate. HUVECs were maintained in EGM-2 MV containing 5% FBS and the cells were stimulated with hypoxia and/or PPARβ/δ agonist for 24 hours. The cells were crosslinked for 10 minutes using 1% paraformaldehyde at the appropriate time thereafter. After neutralization using 0.2 M glycine, cells were recollected, resuspended in SDS lysis buffer (10 mM Tris-HCl, 150 mM NaCl, 1% SDS, 1 mM EDTA; pH 8.0) and fragmented by sonication (Branson, Danbury, CT, USA; 10 minutes). Samples were stored at -80°C before use. To perform ChIP, antibodies against PPARβ/δ, H3K27ac and Pol II were used in combination with magnetic beads (Life Technologies/Thermo Fisher Scientific, South San Francisco, CA, USA). Prepared DNA was quantified using Qubit (Life Technologies/Thermo Fisher Scientific) and more than 10 ng of DNA was processed, as described below. The ChIP primer sequences are listed in Table S3C in Additional file 2.

### ChIP-seq

All of the protocols for Illumina/Solexa sequence preparation, sequencing and quality control were provided by Illumina. Sequences were aligned using human genome NCBI Builder 36 (UCSC hg18) as the reference genome. Non-immunoprecipitated DNA (input DNA) was used as a negative control to define non-specific binding. All uniquely mapped sequences were analyzed by Quantitative Enrichment of Sequence Tags (QuEST) 2.4 software using the default parameters (KDE bandwidth = 30 bp, region size = 300 bp, ChIP seeding fold enrichment = 30, ChIP extension fold enrichment = 3, ChIP-to-background fold enrichment = 3 for PPARβ/δ and HIF1α, KDE bandwidth = 60 bp, region size = 600 bp, ChIP seeding fold enrichment = 30, ChIP extension fold enrichment = 3, ChIP-to-background fold enrichment = 3 for Pol II, KDE bandwidth = 100 bp, region size = 1000 bp, ChIP seeding fold enrichment = 30, ChIP extension fold enrichment = 3, and ChIP-to-background fold enrichment = 3 for H3K27ac, H3K4me1 and H3K4me3) [20]. WIG files were generated with QuEST, which were subsequently used for visualization purposes and for obtaining the average signal profiles. These signals were visualized using Integrated Genome Browser software [58] with normalized profile wig files calculated by QuEST.

### Mapping of ChIP-seq reads

All ChIP-seq analyses were performed using a GAII (Illumina). We duplicated ChIP-seq procedures (Figure S13). For PPARβ/δ, in total 36,607,827 and 36,864,223 (normoxia-DMSO), 38,936,258 and 35,654,581 (normoxia-PPARβ/δ), 36,760,328 and 43,029,618 (hypoxia-DMSO), and 35,058,306 and 43,474,951 (hypoxia-PPARβ/δ) reads were aligned and 76.7% and 78.8% (normoxia-DMSO), 77.6% and 53.7% (normoxia-PPARβ/δ), 74.0% and 79.0% (hypoxia-DMSO), and 78.8% and 79.4% (hypoxia-PPARβ/δ) of the total reads were aligned uniquely to the non-repeating human genomic sequence. For H3K27ac, out of a total of 36,530,846 and 41,560,945 (normoxia-DMSO), 37,346,766 and 44,268,585 (normoxia-PPARβ/δ), 38,316,199 and 38,228,969 (hypoxia-DMSO), 36,900,687 and 41,208,985 (hypoxia-PPARβ/δ) sequence reads, 83.0% and 85.7% (normoxia-DMSO), 85.7% and 85.3% (normoxia-PPARβ/δ), 85.8% and 84.2% (hypoxia-DMSO), and 84.6% and 84.8% (hypoxia-PPARβ/δ) were uniquely mapped. For Pol II, 34,732,013 and 41,475,510 (normoxia-DMSO), 28,155,220 and 42,030,082 (normoxia-PPARβ/δ), 31,755,951 and 43,544,244 (hypoxia-DMSO), and 31,015,638 and 41,895,797 (hypoxia-PPARβ/δ) reads were totally aligned and 81.5% and 80.8% (normoxia-DMSO), 83.0% and 81.4% (normoxia-PPARβ/δ), 83.1% and 81.4% (hypoxia-DMSO), and 83.3% and 81.5% (hypoxia-PPARβ/δ) of the total reads were aligned uniquely to the non-repeating human genomic sequence. Detailed information regarding HIF1α, H3K4me1, H3K4me3 was provided previously [4].

### Motif search

A *de novo* motif search was performed using MEME (version 4.6.1) [59]. All the non-repetitive 100 bp sequences calculated by QuEST were used and analyzed. The MEME parameters were 'distribution of motif occurrences; any number of repetitions, minimum motif width; 6, maximum motif width; 30'. The Weblogo program [60] was used to indicate the motifs. The *E*-value is an estimate of the expected number of motifs with the given log likelihood ratio for the random sequences.

### Motif comparison

The enriched sequence was compared to the known consensus TF motifs using TOMTOM (version 4.8.1) with the motif databases JASPAR and UniPROBE [61]. The Tomtom web application compares an input DNA motif to the elements of a database of known motifs (and their DNA reverse complements). A list of the matching motifs is shown, ranked by *q*-value. The *q*-value is the minimum false discovery rate at which the observed similarity would be deemed significant.

### DNA microarray

HUVECs maintained with EGM-2 MV containing 5% FBS were stimulated with hypoxia and/or the PPARβ/δ agonist for 24 hours. Total cellular RNA was isolated using an RNeasy Micro kit (QIAGEN, Hilden, Germany). Preparation of the cRNA and hybridization of the probe arrays were performed according to the manufacturer’s instructions (Affymetrix, Santa Clara, CA, USA). Affymetrix Genechip Human Genome U133 plus 2.0 arrays containing over 54,000 probe sets were applied. The expression value for each mRNA was obtained by the robust multi-array analysis (RMA) method. To analyze the expression data at the genetic level, the intensity of the signal values was summarized using Entrez Gene ID (normalized to the 75th percentile). Then the gene set probes were filtered on an expression (20.0 to 100.0) percentile. Genes expressed at lower than the 20 percentile in all of the four arrays were eliminated from the analyses. After excluding the gene set probes that did not have gene symbols, the remaining 21,089 genes were used for further analysis. Hierarchical cluster analysis was performed using average linkage and Pearson correlation as a measure of similarity. All analysis was performed with GeneSpring GX 12.5 (Agilent Technologies, Santa Clara, CA, USA). Annotation of the probe numbers and targeted sequences are shown on the Affymetrix web page.

### Binding motif estimation *in silico*

Motif estimation for the binding sites of PPARβ/δ was performed using MatInspector, a software for identification of TF binding sites provided by Genomatix (Munich, Germany)

### Luciferase reporter assay

Transfections were performed with Fugene HD (Roche, Basel, Switzerland). Cells were transfected on 12-well plates at 70 to 80% confluence in Opti-MEM (Life Technologies/Thermo Fisher Scientific) with 0.5 μg of plasmid. Cells were incubated for 24 hours in normal growth medium, then the stimulation was initiated. Luciferase assays were performed 24 hours after stimulation. Luciferase activity was calibrated with the *Renilla* activity. The primers for the site-directed mutagenesis of the HRE and PPRE motifs are shown in Table S3B in Additional file 2.

### Quantitative RT-PCR

Total cellular RNA was isolated using an RNeasy Micro kit (QIAGEN). The cDNA was synthesized from 100 ng of RNA using oligo (dT) primers and the Superscript III kit (Life Technologies/Thermo Fisher Scientific). qPCR was performed on a CFX96 real-time System (BioRad, Hercules, CA, USA) for 45 cycles at an annealing temperature of 60°C. PCRs were carried out using iQ SYBR Green Supermix (BioRad) and a primer concentration of 200 nM, following the manufacturer’s instructions. 18S rRNA was used as the normalizer. The sequences of SYBR Green primers are listed in Table S3A in Additional file 2.

### Western blotting

Protein samples were separated by 10% SDS-PAGE and transferred electrophoretically onto nitrocellulose membranes (Hybond-C; GE Healthcare UK Ltd, Buckinghamshire, England). Membranes were blocked with 5% (w/v) nonfat milk in phosphate-buffered saline containing 0.1% Tween for 1 hour, incubated with antibodies for 1 hour, and detected by chemiluminescence using West Dura extended duration substrate (Thermo Fisher Scientific, Waltham, MA, USA) according to the manufacturer’s protocol.

### Quantitative chromosome conformation capture assay

HUVECs were maintained in EGM-2 MV containing 5% FBS, then the cells were stimulated with hypoxia and/or PPARβ/δ agonist for 24 hours. The assay was performed utilizing the TaqMan 3C Chromosome Conformation Kit (EcoRI; Life Technologies/Thermo Fisher Scientific) according to the manufacturer’s instructions, with certain modifications. Briefly, HUVECs were crosslinked with 1% paraformaldehyde for 10 minutes. The reaction was stopped by the addition of 125 mM glycine for 5 minutes. Nuclei were re-suspended with restriction enzyme buffer. Samples were treated with 400 U of *Sau3AI* at 37°C for 16 hours. After enzyme digestion, the samples were diluted with ligation buffer and treated with DNA ligase at 16°C for 1 hour. Samples were finally reverse crosslinked and purified. All primers and TaqMan probes are shown in Table S3D in Additional file 2 and the positions of the primers are shown in Figure 5A. We used 50 ng of DNA for the 3C assay. In addition, to normalize template loading, an input normalization assay in the 3C kit was used according to the reported protocol with some modification [62]. Two human BAC clones (RP11-995 M24, RP11-978 J4) were used to normalize the levels of 3C products that may have been PCR amplified with different primer efficiencies, as mentioned previously [62,63].

### Gene knockdown by siRNA

EGM-2 MV used for HUVEC culture was replaced with Opti-MEM culture medium, and the cells were transfected with stealth RNA interference for HIF1α (Life Technologies/Thermo Fisher Scientific, HSS104774 and HSS104775) and PPARβ/δ (Life Technologies/Thermo Fisher Scientific, HSS108293 and HSS108294) at a concentration of 10 nM using Lipofectamine RNAiMax reagent (Life Technologies/Thermo Fisher Scientific). After 6 hours, the culture medium was changed back to EGM-2 MV. After another 24 hours, cells were stimulated with hypoxia and/or the PPARβ/δ agonist. The knockdown efficiencies of HIF1α and PPARβ/δ were validated by qRT-PCR (CFX96, BioRad) using the same primers described above (Table S3A in Additional file 2).

### Cell migration assay

We plated 150,000 HUVECs in a 35 mm type I collagen-coated culture plate (IWAKI, Asahi glass, Tokyo, Japan) and incubated the cells under usual conditions for 48 hours. After making a scratch in the cell monolayer using a 5 ml serological pipette, EGM-2 MV was replaced with EBM-2 culture medium for Figure 1 or EGM-2 MV culture medium for Figure S6 in Additional file 1, and then the stimulation was initiated or recombinant ANGPTL4 was added. The first image of the scratch was acquired at this time. After 8 hours, an image was obtained at exactly the same place as the first image. The images acquired for each sample were analyzed quantitatively using the software Image J (NIH) and the migration areas were calculated.

### Statistical analysis

*P*-values were calculated using two-tailed unpaired Student’s *t*-test. *P* < 0.05 was considered significant.

### Availability of data

Data are available from the Gene Expression Omnibus [64]. The ChIP-seq datasets are available at accession numbers GSE38555 and GSE50144 and the microarray analysis is accession GSE50378.

## Abbreviations

3C: chromatin conformation capture; ANGPTL: angiopoietin-like; ChIP: chromatin immunoprecipitation; FBS: fetal bovine serum; HIF: hypoxia-inducible factor; HRE: hypoxia response element; HUVEC: human umbilical vein endothelial cell; PCR: polymerase chain reaction; Pol II: RNA polymerase II; PPAR: peroxisome proliferator-activated receptor; PPRE: PPAR-response element; qRT-PCR: quantitative RT-PCR; siRNA: small interfering RNA; TF: transcription factor; TGF: transforming growth factor; TSS: transcription start site; UTR: untranslated region; VEGF: vascular endothelial growth factor.

## Competing interests

Hirofumi Aruga, Shoulian Dong and Junko Stevens are employees of Thermo Fisher Scientific, which supplied the materials for Chromosome Conformation Capture (3C) and qPCR assays. Takashi Maejima is an employee of Kowa Company Ltd.

## Authors’ contributions

TI designed the studies, carried out the molecular genetic studies, and drafted the manuscript. TK, GL, HP, SY, ST, and XR analyzed genome-wide data. TT, YK, TF, HA, MN, YR, and TK designed the studies. IM, MK, AT, TM, JS, AS, KK, HA, SD, and JS prepared the reagents and performed the experiments. YW designed the studies and drafted the manuscript. All authors read and approved the final manuscript.

## Authors’ information

Toshiya Tanaka partial correspondence for PPARβ/δ antibody.

## Supplementary Material

Additional file 1: Figure S1 Scatter plot of the genes affected by PPARβ/δ agonist or hypoxia treatment in endothelial cells. **Figure S2.** Classification of the hierarchical entity tree for the samples. **Figure S3.** Scatter plot of the genes induced by the PPARβ/δ agonist and/or hypoxia. **Figure S4.** Real time PCR of *ANGPTL4* with primers recognizing intron-exon junctions. **Figure S5.** Western blotting of ANGPTL4 under the four conditions. **Figure S6.** Endothelial cell migration is enhanced by *ANGPTL4*. **Figure S7.** Genome-wide analysis of PPARβ/δ and/or hypoxia target genes. **Figure S8.** Efficiency of the siRNA-mediated knockdown of PPARβ/δ and HIF1α. **Figure S9.** Real time PCR of *ANGPTL4* with siPPARβ/δ under PPARβ/δ agonist stimulation, with siHIF1α under hypoxia, or with siHIF1α and siPPARβ/δ under hypoxia and the PPARβ/δ agonist stimulations. **Figure S10.** Distribution of the H3K27ac binding regions under the four conditions. **Figure S11.** Sequence of the 3C product. **Figure S12.** ChIP-PCR of Pol II under the four conditions. **Figure S13.** ChIP-seq of PPARβ/δ, H3K27ac and Pol II in duplicate. Click here for file

Additional file 2: Table S1 Common and PPARβ/δ agonist target genes. **Table S2.** Hypoxia target genes. **Table S3.** Primers used. Click here for file
