# Supplementary material for: Cross-enhancement of ANGPTL4 transcription by HIF1 alpha and PPAR beta/delta is the result of the conformational proximity of two response elements
Source: Genome Biol. 2014 Apr 10;15(4):R63. doi: 10.1186/gb-2014-15-4-r63 (PMC4053749; doi:10.1186/gb-2014-15-4-r63)

Inoue_Fig. S1

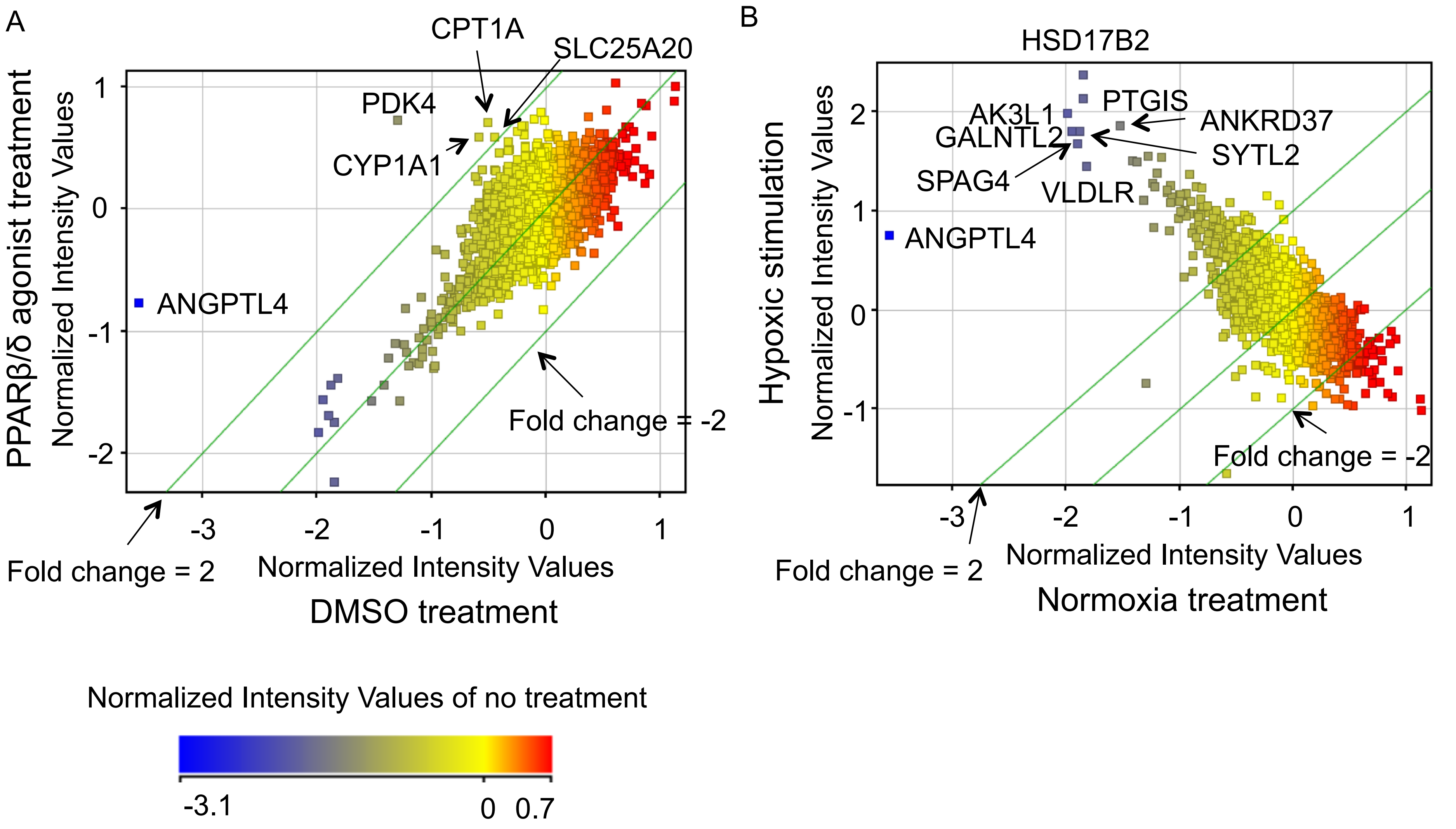
A
CPT1A
SLC25A20
PDK4
CYP1A1
ANGPTL4
PPARβ/δ agonist treatment
Normalized Intensity Values
Fold change = -2
Fold change = 2
Normalized Intensity Values
DMSO treatment
1
0
-1
-2
-3
-2
-1
0
1
B
HSD17B2
PTGIS
AK3L1
ANKRD37
GALNTL2
SYTL2
SPAG4
VLDLR
ANGPTL4
Hypoxic stimulation
Normalized Intensity Values
2
1
0
-1
-3
-2
-1
0
1
Fold change = -2
Fold change = 2
Normalized Intensity Values
Normoxia treatment
Normalized Intensity Values of no treatment
-3.1
0
0.7

Inoue_Fig. S2

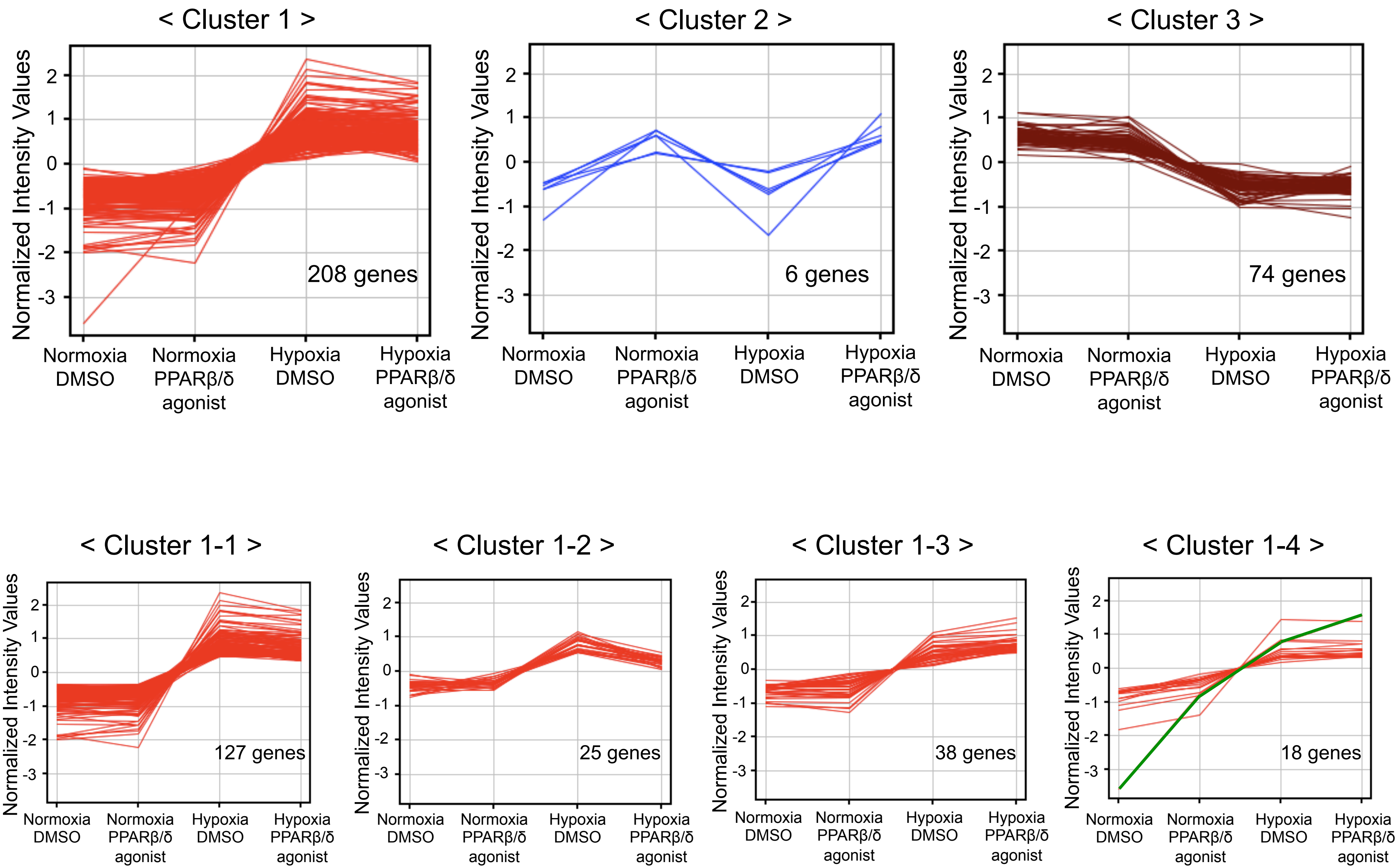

< Cluster 1 >
208 genes
< Cluster 2 >
6 genes
< Cluster 3 >
74 genes
< Cluster 1-1 >
127 genes
< Cluster 1-2 >
25 genes
< Cluster 1-3 >
38 genes
< Cluster 1-4 >
18 genes
Normalized Intensity Values
2
1
0
-1
-2
-3
Normoxia DMSO
Normoxia PPARβ/δ agonist
Hypoxia DMSO
Hypoxia PPARβ/δ agonist

Inoue_Fig. S3

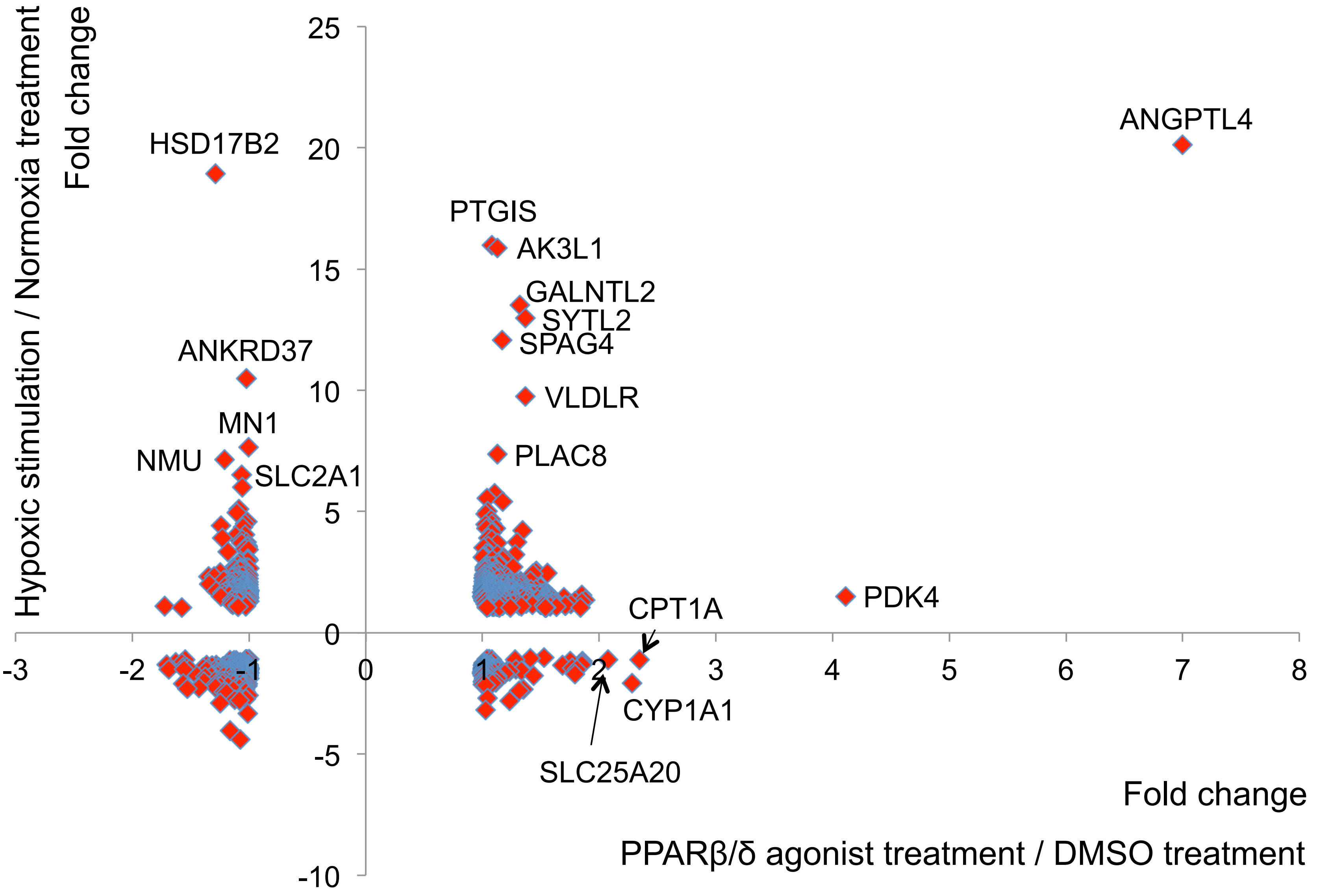
Hypoxic stimulation / Normoxia treatment
Fold change
25
20
15
10
5
0
-5
-10
-3
-2
-1
0
1
2
3
4
5
6
7
8
HSD17B2
ANKRD37
MN1
NMU
SLC2A1
PTGIS
AK3L1
GALNTL2
SYTL2
SPAG4
VLDLR
PLAC8
ANGPTL4
PDK4
CPT1A
CYP1A1
SLC25A20
Fold change
PPARβ/δ agonist treatment / DMSO treatment

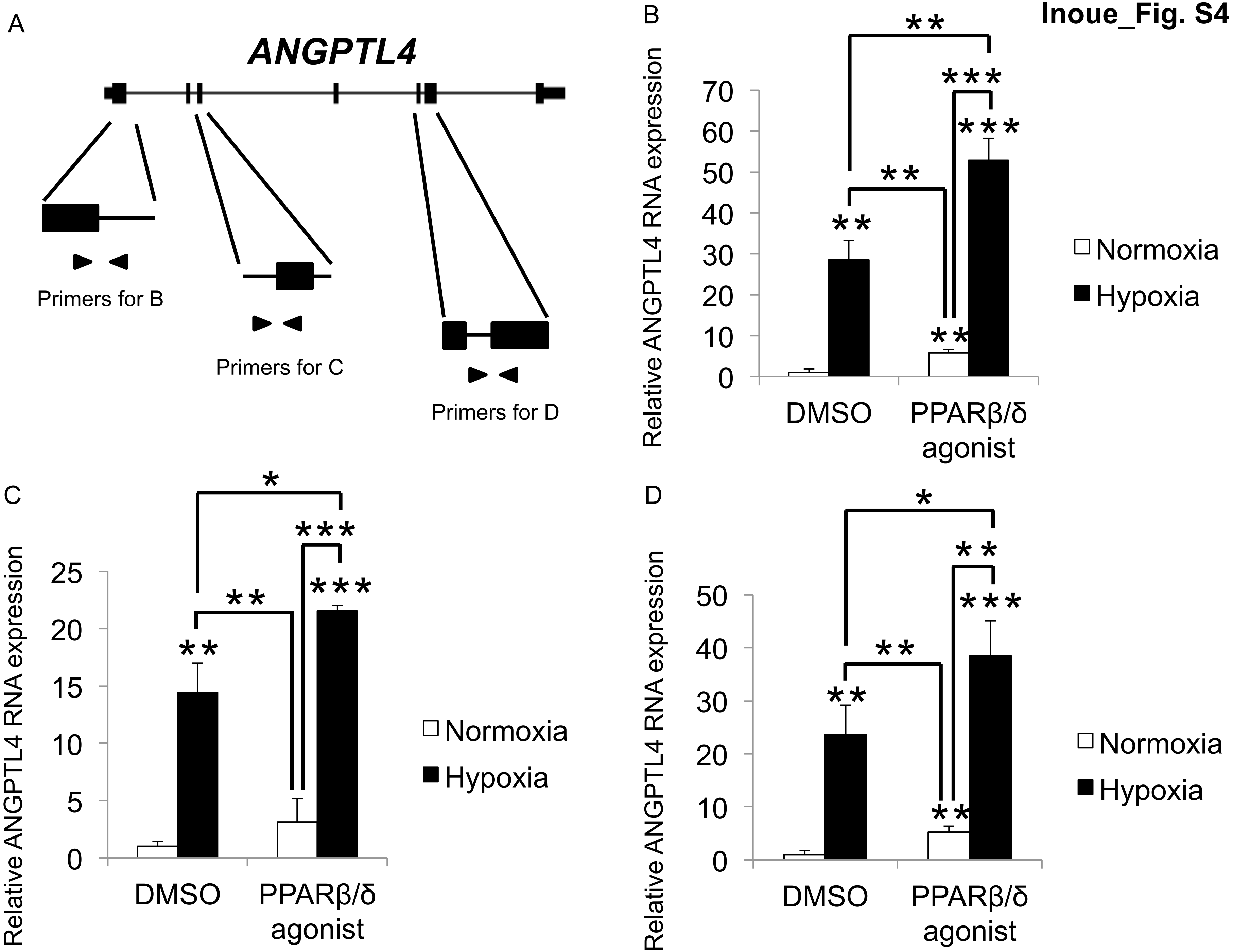
Inoue_Fig. S4
A
ANGPTL4
Primers for B
Primers for C
Primers for D
B
Relative ANGPTL4 RNA expression
0
10
20
30
40
50
60
70
DMSO
PPARβ/δ agonist
Normoxia
Hypoxia
C
Relative ANGPTL4 RNA expression
0
5
10
15
20
25
DMSO
PPARβ/δ agonist
Normoxia
Hypoxia
D
Relative ANGPTL4 RNA expression
0
10
20
30
40
50
DMSO
PPARβ/δ agonist
Normoxia
Hypoxia

Inoue_Fig. S5

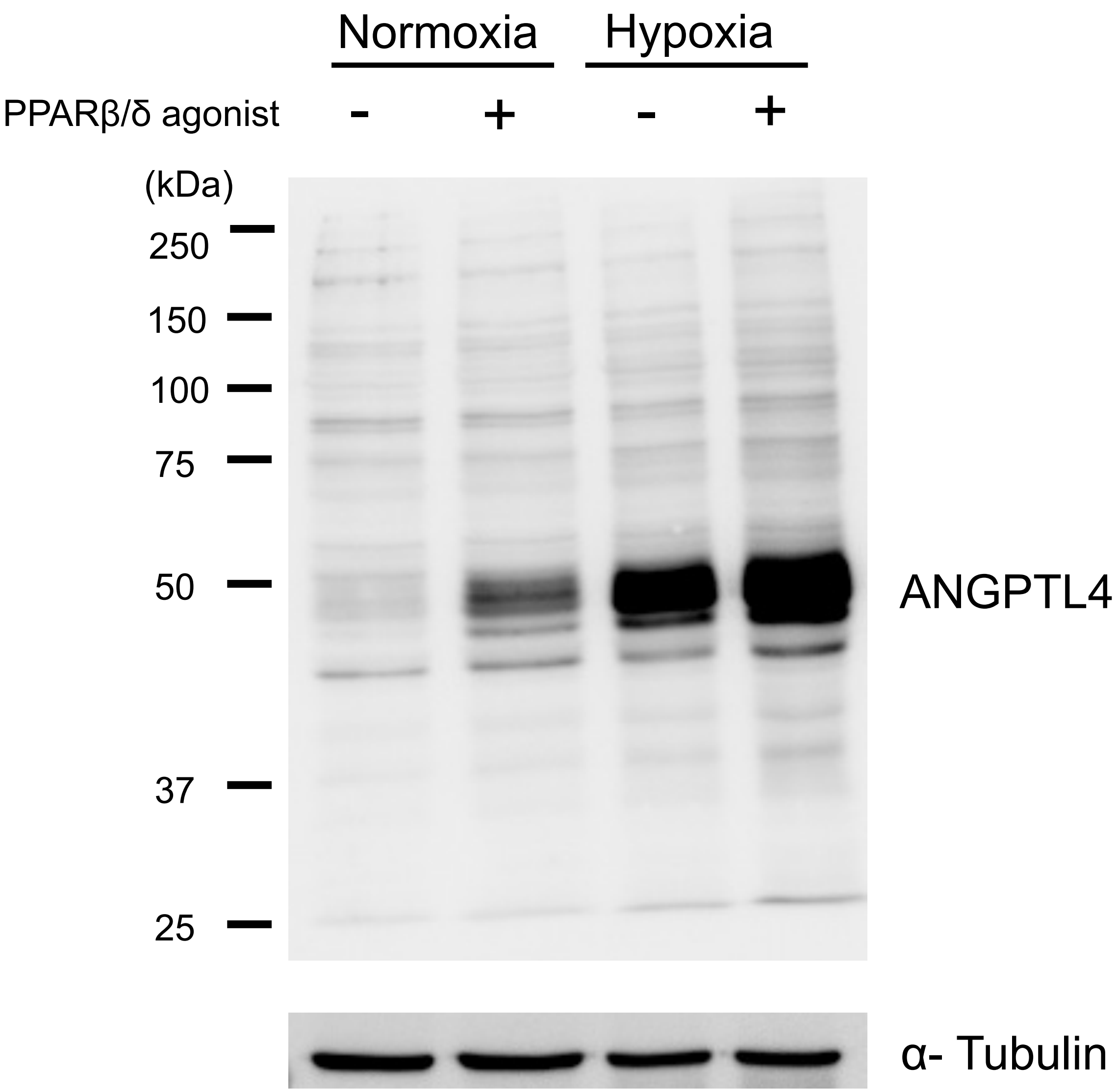
Normoxia
Hypoxia
PPARβ/δ agonist
-
+
-
+
(kDa)
250
150
100
75
50
37
25
ANGPTL4
α- Tubulin

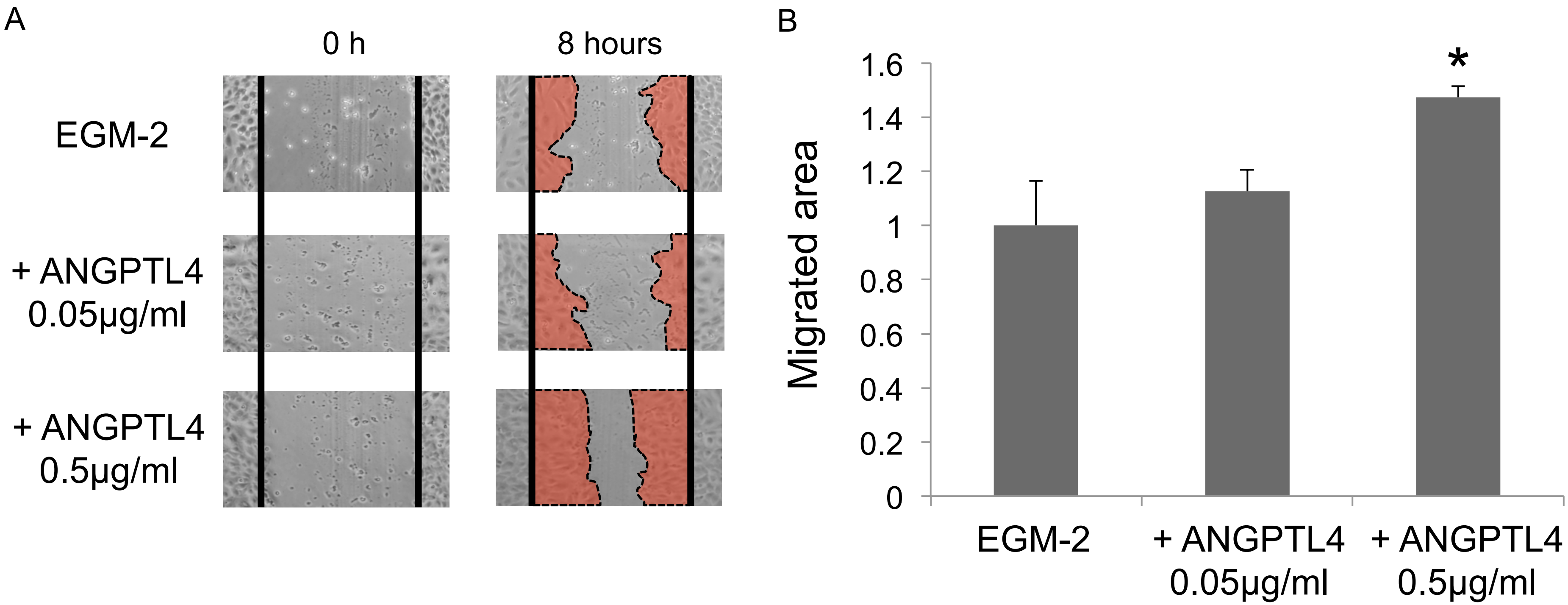
A
0 h
8 hours
EGM-2
+ ANGPTL4 0.05μg/ml
+ ANGPTL4 0.5μg/ml
B
Migrated area
1.6
1.4
1.2
1
0.8
0.6
0.4
0.2
0
*
EGM-2
+ ANGPTL4 0.05μg/ml
+ ANGPTL4 0.5μg/ml

Inoue_Fig. S7

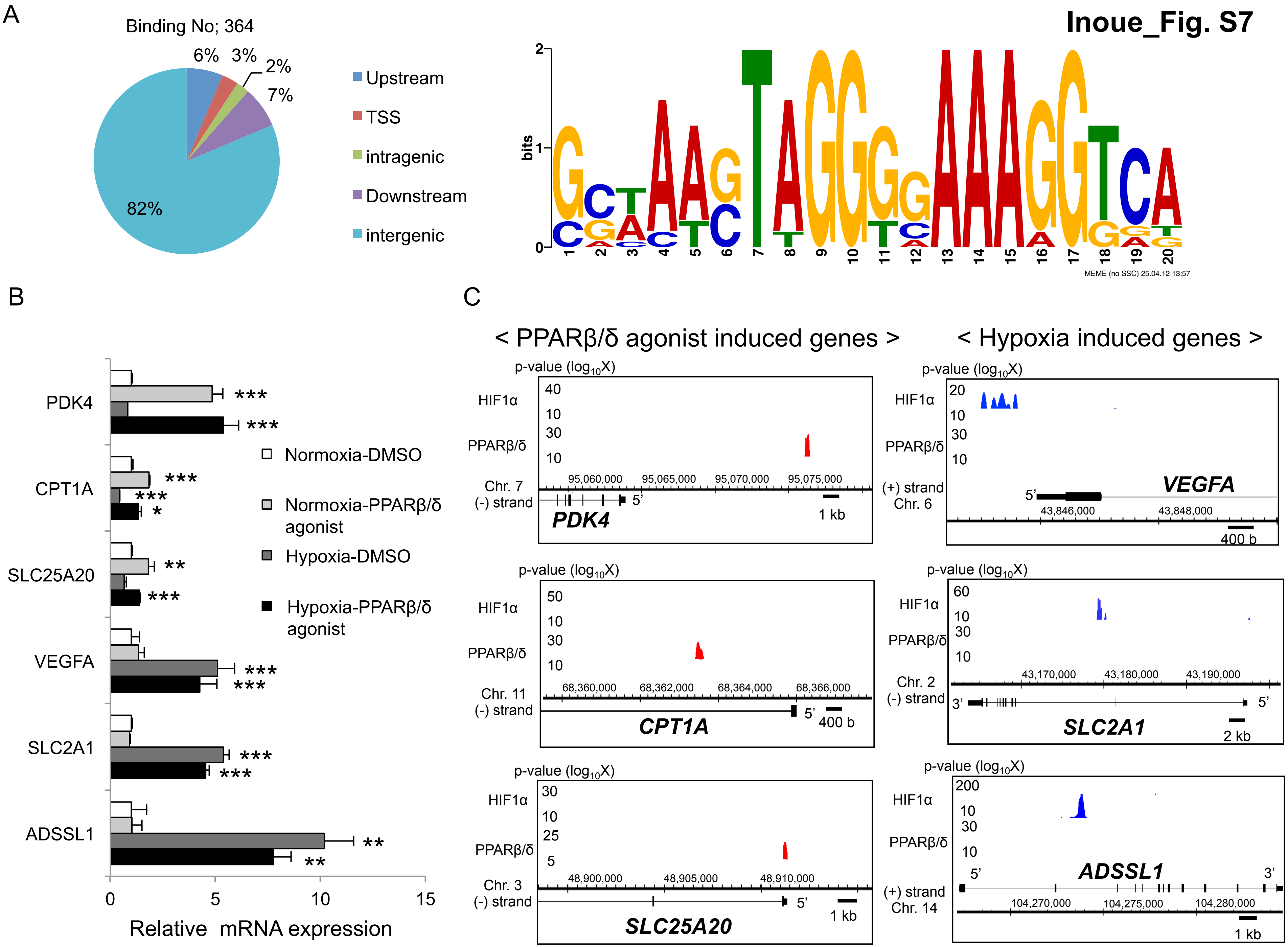
A
Binding No; 364
6%
3%
2%
7%
82%
Upstream
TSS
intragenic
Downstream
intergenic
bits
MEME (no SSC) 25.04.12 13:57
B
PDK4
CPT1A
SLC25A20
VEGFA
SLC2A1
ADSSL1
Normoxia-DMSO
Normoxia-PPARβ/δ agonist
Hypoxia-DMSO
Hypoxia-PPARβ/δ agonist
Relative mRNA expression
C
< PPARβ/δ agonist induced genes >
< Hypoxia induced genes >
p-value (log10X)
HIF1α
PPARβ/δ
Chr. 7
(-) strand
PDK4
1 kb
Chr. 11
(-) strand
CPT1A
400 b
Chr. 3
(-) strand
SLC25A20
(+) strand
Chr. 6
VEGFA
Chr. 2
(-) strand
SLC2A1
2 kb
(+) strand
Chr. 14
ADSSL1

Inoue_Fig. S8

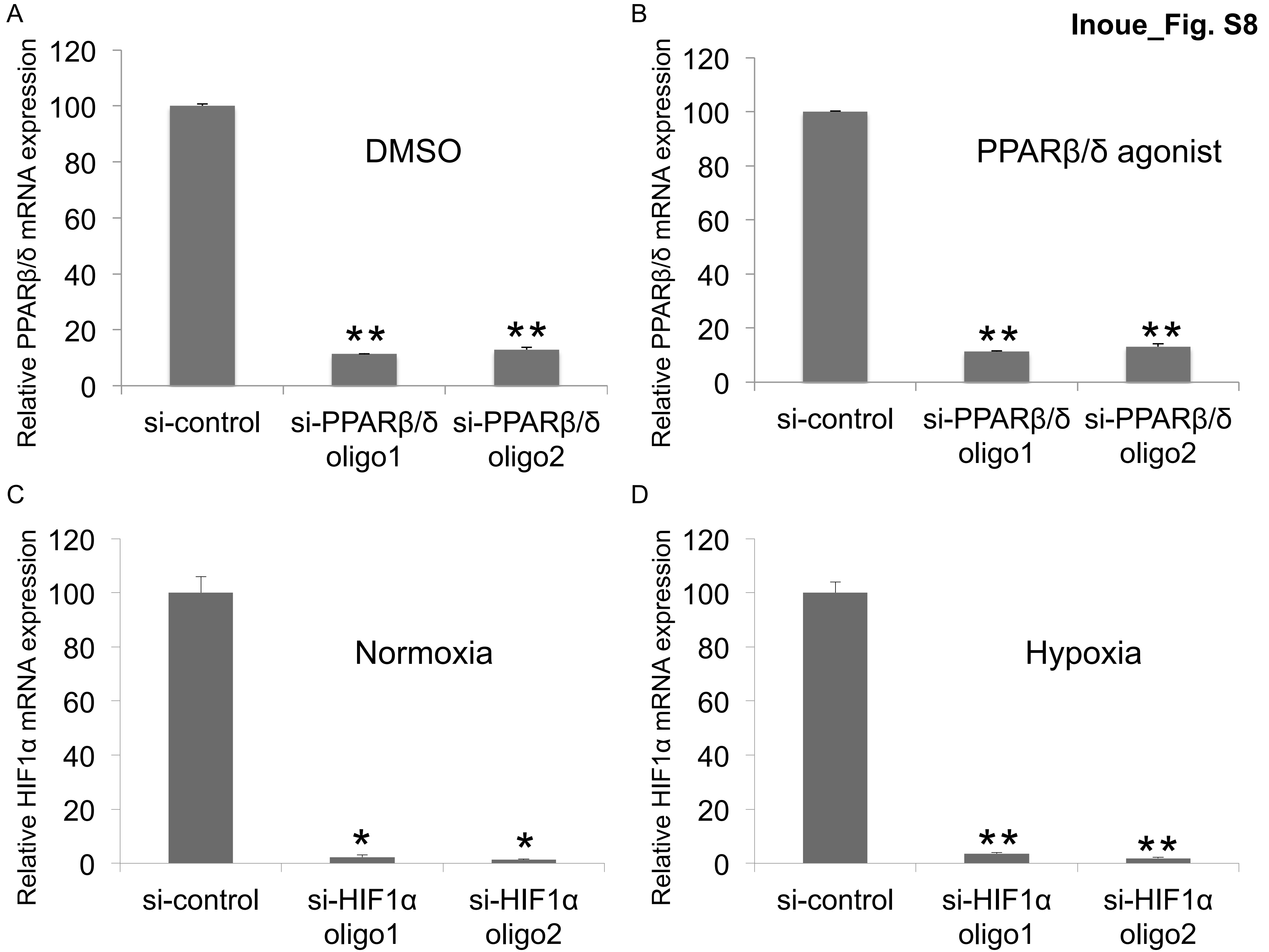
A
Relative PPARβ/δ mRNA expression
120
100
80
60
40
20
0
DMSO
**
**
si-control
si-PPARβ/δ oligo1
si-PPARβ/δ oligo2
B
Relative PPARβ/δ mRNA expression
120
100
80
60
40
20
0
PPARβ/δ agonist
**
**
si-control
si-PPARβ/δ oligo1
si-PPARβ/δ oligo2
C
Relative HIF1α mRNA expression
120
100
80
60
40
20
0
Normoxia
*
*
si-control
si-HIF1α oligo1
si-HIF1α oligo2
D
Relative HIF1α mRNA expression
120
100
80
60
40
20
0
Hypoxia
**
**
si-control
si-HIF1α oligo1
si-HIF1α oligo2

Inoue_Fig. S9

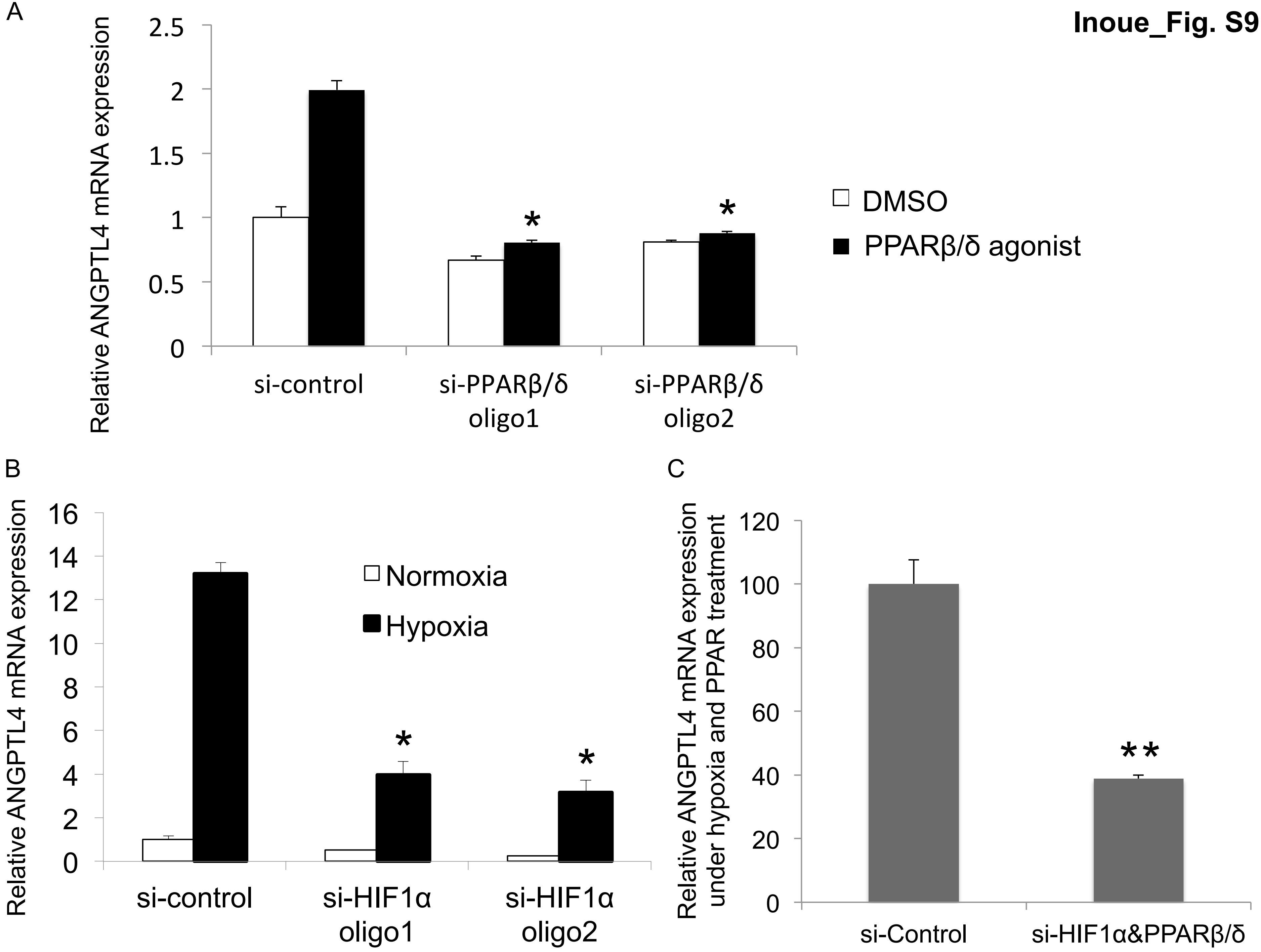
A
Relative ANGPTL4 mRNA expression
2.5
2
1.5
1
0.5
0
si-control
si-PPARβ/δ oligo1
si-PPARβ/δ oligo2
*
*
DMSO
PPARβ/δ agonist
B
Relative ANGPTL4 mRNA expression
16
14
12
10
8
6
4
2
0
Normoxia
Hypoxia
*
*
si-control
si-HIF1α oligo1
si-HIF1α oligo2
C
Relative ANGPTL4 mRNA expression under hypoxia and PPAR treatment
120
100
80
60
40
20
0
**
si-Control
si-HIF1α&PPARβ/δ

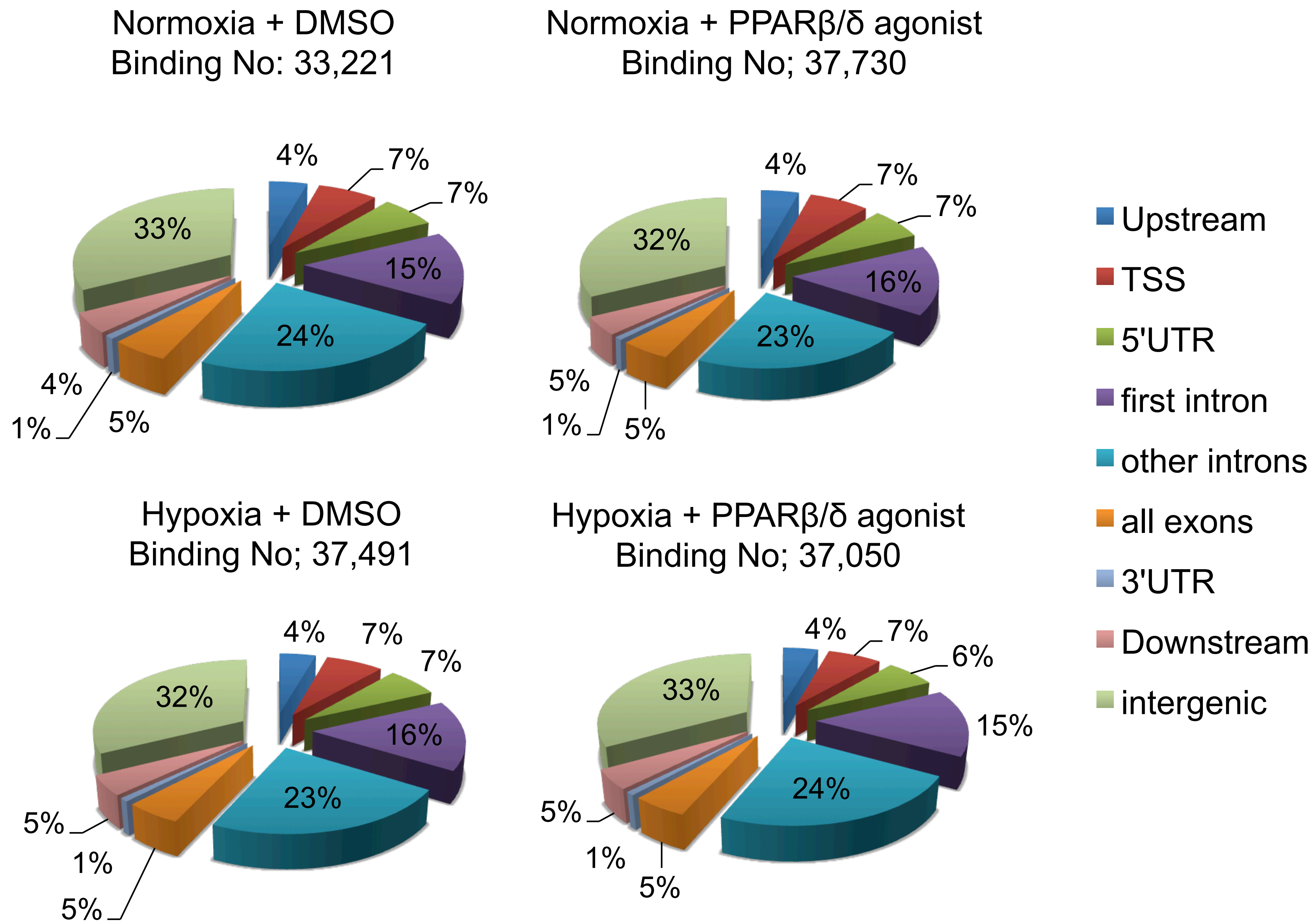
Normoxia + DMSO
Binding No: 33,221
4%
7%
7%
33%
15%
24%
4%
1%
5%
Normoxia + PPARβ/δ agonist
Binding No; 37,730
4%
7%
7%
32%
16%
23%
5%
1%
5%
Hypoxia + DMSO
Binding No; 37,491
4%
7%
7%
32%
16%
23%
5%
1%
5%
Hypoxia + PPARβ/δ agonist
Binding No; 37,050
4%
7%
6%
33%
15%
24%
5%
1%
5%
Upstream
TSS
5'UTR
first intron
other introns
all exons
3'UTR
Downstream
intergenic

Inoue_Fig. S11

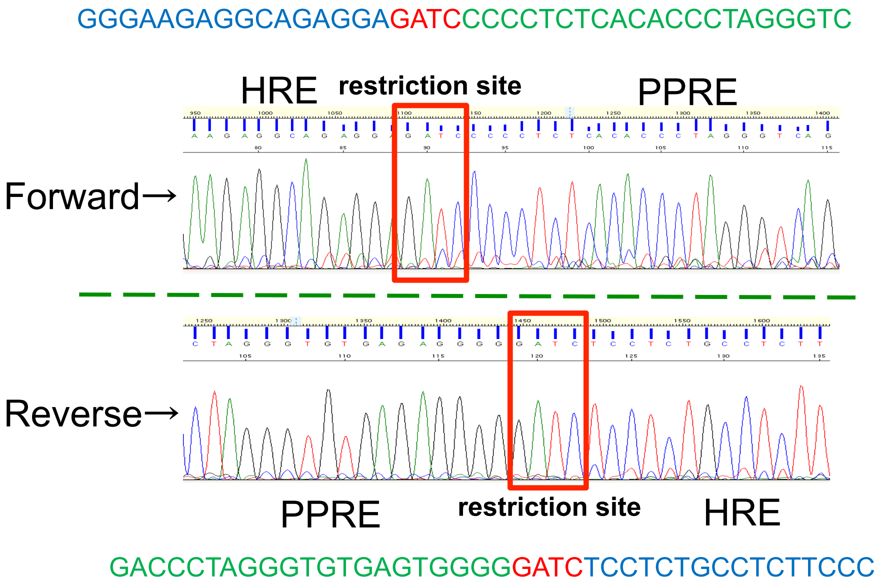

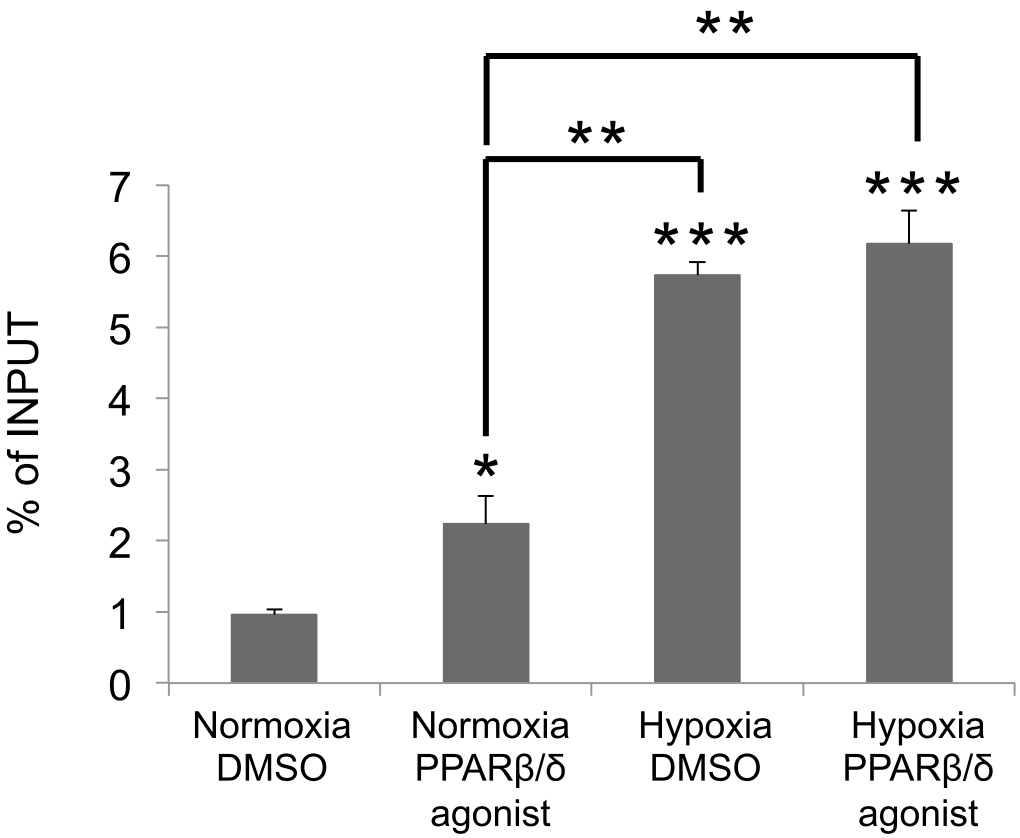
**
**
***
***
*
7
6
5
4
3
2
1
0
% of INPUT
Normoxia DMSO
Normoxia PPARβ/δ agonist
Hypoxia DMSO
Hypoxia PPARβ/δ agonist

Inoue_Fig. S13

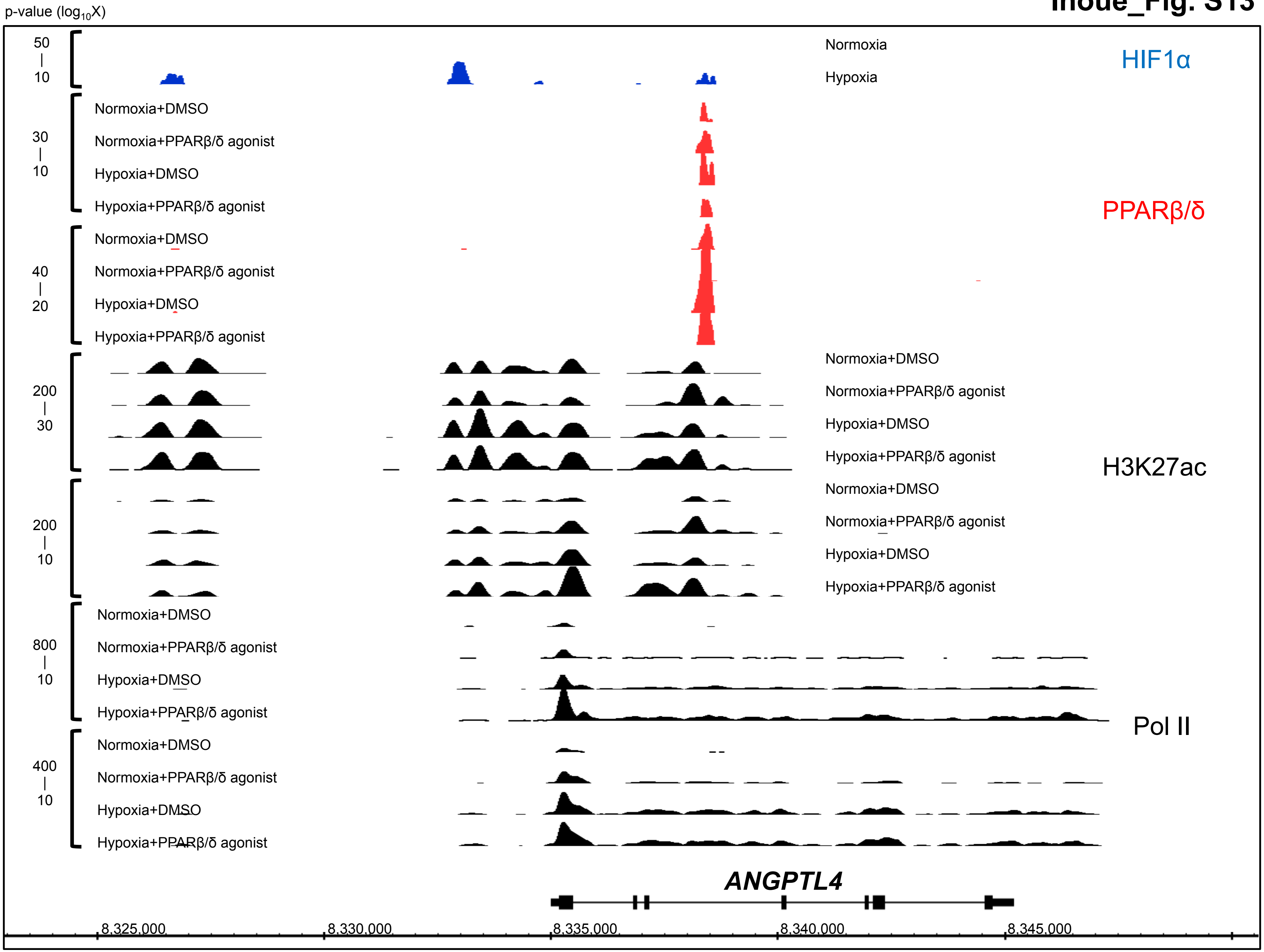

Supplement: Additional file 1: Figure S1 — Scatter plot of the genes affected by PPARβ/δ agonist or hypoxia treatment in endothelial cells. Figure S2. Classification of the hierarchical entity tree for the samples. Figure S3. Scatter plot of the genes induced by the PPARβ/δ agonist and/or hypoxia. Figure S4. Real time PCR of ANGPTL4 with primers recognizing intron-exon junctions. Figure S5. Western blotting of ANGPTL4 under the four conditions. Figure S6. Endothelial cell migration is enhanced by ANGPTL4. Figure S7. Genome-wide analysis of PPARβ/δ and/or hypoxia target genes. Figure S8. Efficiency of the siRNA-mediated knockdown of PPARβ/δ and HIF1α. Figure S9. Real time PCR of ANGPTL4 with siPPARβ/δ under PPARβ/δ agonist stimulation, with siHIF1α under hypoxia, or with siHIF1α and siPPARβ/δ under hypoxia and the PPARβ/δ agonist stimulations. Figure S10. Distribution of the H3K27ac binding regions under the four conditions. Figure S11. Sequence of the 3C product. Figure S12. ChIP-PCR of Pol II under the four conditions. Figure S13. ChIP-seq of PPARβ/δ, H3K27ac and Pol II in duplicate. [file gb-2014-15-4-r63-S1.pdf]
