# Supplementary material for: Cross-enhancement of ANGPTL4 transcription by HIF1 alpha and PPAR beta/delta is the result of the conformational proximity of two response elements
Source: Genome Biol. 2014 Apr 10;15(4):R63. doi: 10.1186/gb-2014-15-4-r63 (PMC4053749; doi:10.1186/gb-2014-15-4-r63)
Supplement: Additional file 2: Table S1 — Common and PPARβ/δ agonist target genes. Table S2. Hypoxia target genes. Table S3. Primers used. [file gb-2014-15-4-r63-S2.pdf]

**Supplemental Table S1. Common & PPARβ/δ agonist target genes**

| Probe ID | Entrez Gene | Gene Symbol | Microarray analysis (fold change) | | |
|---|---|---|---|---|---|
| | | | With PPARβ/δ agonist | Under Hypoxia | Under both stimulations |
| Common target genes (The genes induced by the PPARβ/δ agonist and hypoxia) | | | | | |
| 221009_s_at, 223333_s_at | 51129 | ANGPTL4 | 7.0 | 20.1 | 35.3 |
| 225283_at | 91947 | ARRDC4 | 1.6 | 2.5 | 2.5 |
| 227443_at | 286343 | LURAP1L | 1.5 | 1.9 | 2.2 |
| PPARβ/δ agonist target genes (The genes induced by the PPARβ/δ agonist, but not hypoxia) | | | | | |
| 205960_at, 225207_at 1562321_at | 5166 | PDK4 | 4.1 | 1.49 | 4.3 |
| 203633_at, 203634_s_at 210687_at, 210688_s_at | 1374 | CPT1A | 2.3 | -1.1 | 2.0 |
| 205749_at | 1543 | CYP1A1 | 2.3 | -2.1 | 3.3 |
| 203658_at | 788 | SLC25A20 | 2.1 | -1.1 | 1.9 |
| 209122_at, 215895_x_at | 123 | ADFP | 1.8 | 1.3 | 2.1 |
| 212665_at, 1556829_at | 25976 | TIPARP | 1.6 | 1.2 | 2.1 |

**Supplemental Table S2. Hypoxia target genes**

| Probe ID | Entrez Gene | Gene Symbol | Under Hypoxia (fold change) | With PPARβ/δ agonist (fold change) | Under both stimulations (fold change) |
|---|---|---|---|---|---|
| 221009_s_at,223333_s_at | 51129 | ANGPTL4 | 20.10903 | 6.998707 | 35.276196 |
| 204818_at | 3294 | HSD17B2 | 18.932432 | -1.2867024 | 13.1734 |
| 208131_s_at,210702_s_at,211892_s_at | 5740 | PTGIS | 15.9720545 | 1.0815737 | 12.081443 |
| 204347_at,204348_s_at,225342_at,230630_at | 205 | AK3L1 | 15.858082 | 1.1263087 | 14.116184 |
| 228501_at,236361_at,239461_at | 117248 | GALNTL2 | 13.51196 | 1.3196096 | 11.324924 |
| 220613_s_at,225496_s_at,232914_s_at | 54843 | SYTL2 | 12.964958 | 1.370452 | 9.967332 |
| 219888_at | 6676 | SPAG4 | 12.076656 | 1.1672696 | 12.134428 |
| 227337_at,242548_x_at | 353322 | ANKRD37 | 10.491178 | -1.0201494 | 8.359237 |
| 209822_s_at | å | VLDLR | 9.754207 | 1.3684555 | 9.247862 |
| 205330_at | 4330 | MN1 | 7.648098 | -1.0045148 | 7.219291 |
| 219014_at | 51316 | PLAC8 | 7.3558273 | 1.1268914 | 6.039248 |
| 206023_at | 10874 | NMU | 7.153032 | -1.2117007 | 5.9229097 |
| 201249_at,201250_s_at | 6513 | SLC2A1 | 6.5150785 | -1.0630188 | 5.0163207 |
| 226415_at | 57687 | VAT1L | 6.016297 | -1.0552046 | 5.2956805 |
| 202912_at | 133 | ADM | 5.742194 | 1.1007686 | 5.0649753 |
| 231798_at | 9241 | NOG | 5.5532675 | 1.0385559 | 5.260714 |
| 219410_at | 55076 | TMEM45A | 5.413069 | 1.1743897 | 5.3599772 |
| 213338_at | 25907 | TMEM158 | 5.0977197 | -1.0890547 | 4.505342 |
| 227488_at,228235_at,229784_at | 84848 | MGC16121 | 5.017606 | 1.0444225 | 4.476229 |
| 201313_at | 2026 | ENO2 | 4.9443874 | -1.1037586 | 4.681068 |
| 205013_s_at | 135 | ADORA2A | 4.902788 | 1.0262256 | 3.493641 |
| 221272_s_at,223125_s_at,223126_s_at,223127_s_at,237098_at | 81563 | C1orf21 | 4.6748276 | 1.076107 | 4.1597204 |
| 202022_at | 230 | ALDOC | 4.576794 | -1.0135684 | 4.2470136 |
| 224797_at | 57561 | ARRDC3 | 4.5106773 | -1.035341 | 6.1319313 |
| 202364_at | 4601 | MXI1 | 4.500933 | 1.0816774 | 3.7139523 |
| 224657_at | 54206 | ERRFI1 | 4.4689636 | 1.0278349 | 3.8591936 |
| 202887_s_at | 54541 | DDIT4 | 4.4260054 | -1.2394512 | 3.9378142 |

| | | | | | |
|---|---|---|---|---|---|
| 204298_s_at,213640_s_at,215446_s_at | 4015 | LOX | 4.366602 | -1.0497099 | 3.727407 |
| 213942_at,226869_at | 1953 | MEGF6 | 4.3172774 | 1.1134712 | 3.6299882 |
| 226325_at,238259_at | 122622 | ADSSL1 | 4.2958794 | 1.0620162 | 3.7998059 |
| 201506_at | 7045 | TGFBI | 4.294224 | 1.0306468 | 3.1867814 |
| 204595_s_at,204596_s_at,204597_x_at | 6781 | STC1 | 4.216043 | 1.3428713 | 4.1274953 |
| 204239_s_at | 4826 | NNAT | 4.121432 | 1.0805768 | 2.8460157 |
| 201008_s_at,201009_s_at,201010_s_at | 10628 | TXNIP | 4.0431147 | -1.0351532 | 3.542655 |
| 1559034_at | 284759 | SIRPB2 | 3.9883773 | -1.1183425 | 4.5273523 |
| 207543_s_at,243335_at | 5033 | P4HA1 | 3.917186 | 1.049873 | 3.6096933 |
| 214978_s_at | 8497 | PPFIA4 | 3.900043 | -1.2310604 | 5.1829367 |
| 201968_s_at | 5236 | PGM1 | 3.8619025 | 1.0797421 | 3.7957754 |
| 204790_at | 4092 | SMAD7 | 3.85889 | -1.0582205 | 3.1265576 |
| 213715_s_at | 256949 | KANK3 | 3.7383533 | 1.299243 | 3.5162156 |
| 218541_s_at | 56892 | C8orf4 | 3.7312787 | -1.0198231 | 3.9656029 |
| 202497_x_at,202498_s_at,202499_s_at,216236_s_at,222088_s_at | 6515 | SLC2A3///SLC2A14 | 3.7133472 | -1.0656692 | 3.134845 |
| 209201_x_at,211919_s_at,217028_at | 7852 | CXCR4 | 3.7048063 | 1.1289425 | 3.165013 |
| 200632_s_at | 10397 | NDRG1 | 3.5996413 | -1.0141298 | 2.9991639 |
| 204926_at,210511_s_at | 3624 | INHBA | 3.5783617 | 1.0507447 | 2.880707 |
| 205920_at,205921_s_at,211030_s_at,228754_at,228756_at | 6533 | SLC6A6 | 3.5425673 | -1.0106163 | 3.222889 |
| 216236_s_at,222088_s_at | 144195 | SLC2A3///SLC2A14 | 3.5091033 | 1.0078244 | 2.8309 |
| 232504_at | 285628 | LOC285628 | 3.4528813 | -1.03641 | 3.2175832 |
| 221578_at,49306_at,226436_at,237294_at | 83937 | RASSF4 | 3.4363368 | -1.0020229 | 3.1121616 |
| 203953_s_at,203954_x_at | 1365 | CLDN3 | 3.344086 | -1.1825979 | 2.8395362 |
| 201848_s_at,201849_at | 664 | BNIP3 | 3.2841182 | 1.0305194 | 3.7509923 |
| 210512_s_at,210513_s_at,211527_x_at,212171_x_at | 7422 | VEGFA | 3.2560515 | 1.059625 | 3.0206482 |
| 209795_at | 969 | CD69 | 3.2351506 | 1.2784197 | 2.019427 |
| 204284_at,240187_at | 5507 | PPP1R3C | 3.2231414 | 1.0481321 | 2.5696115 |
| 207255_at,209894_at,211354_s_at,211355_x_at,211356_x_at,1556919_a | 3953 | LEPR | 3.2162414 | 1.1283901 | 3.0903177 |
| 218849_s_at | 10848 | PPP1R13L | 3.15144 | 1.0277561 | 2.8661413 |
| 223276_at | 85027 | MST150 | 3.1432748 | 1.0138717 | 2.1463187 |
| 212689_s_at,242758_x_at | 55818 | KDM3A | 3.1113708 | 1.0020725 | 3.193421 |

| | | | | | |
|---|---|---|---|---|---|
| 219702_at | 10761 | PLAC1 | 3.1000793 | 1.1821946 | 2.2554028 |
| 202796_at,235128_at,235914_at | 11346 | SYNPO | 3.0607767 | -1.0115477 | 2.4617748 |
| 221031_s_at | 81575 | APOLD1 | 3.0205743 | 1.1764495 | 2.8127327 |
| 219232_s_at,222847_s_at | 112399 | EGLN3 | 3.019163 | 1.0871795 | 2.35681 |
| 202733_at | 8974 | P4HA2 | 3.0142086 | -1.0027268 | 2.751664 |
| 204881_s_at,221765_at,224967_at | 7357 | UGCG | 2.998255 | -1.0145199 | 2.6139965 |
| 201169_s_at,201170_s_at | 8553 | BHLHE40 | 2.965117 | -1.0116216 | 2.593799 |
| 201147_s_at,201148_s_at,201149_s_at,201150_s_at | 7078 | TIMP3 | 2.9071414 | -1.0830353 | 2.3944306 |
| 228362_s_at,229390_at,229391_s_at,229543_at,229915_at | 441168 | FAM26F | 2.8846965 | 1.1309612 | 2.5181847 |
| 220388_at,222245_s_at | 80307 | FER1L4 | 2.8095615 | 1.1387254 | 3.1754975 |
| 228770_at | 115330 | GPR146 | 2.7955272 | -1.0950174 | 2.4899452 |
| 221478_at,221479_s_at | 665 | BNIP3L | 2.791297 | 1.0345457 | 2.8819861 |
| 233565_s_at | 27111 | SDCBP2 | 2.7470639 | 1.096678 | 2.621835 |
| 210715_s_at | 10653 | SPINT2 | 2.7400997 | 1.0381677 | 2.3365803 |
| 201041_s_at,201044_x_at | 1843 | DUSP1 | 2.739735 | 1.062241 | 2.3276436 |
| 203973_s_at,213003_s_at,213006_at | 1052 | CEBPD | 2.7347922 | 1.0870358 | 2.3310966 |
| 203066_at | 51363 | GALNAC4S-6ST | 2.709463 | 1.2635218 | 3.0513194 |
| 213900_at | 9413 | C9orf61 | 2.7082953 | 1.0218042 | 2.0367332 |
| 207574_s_at,209304_x_at,209305_s_at,213560_at | 4616 | GADD45B | 2.6910226 | -1.0672487 | 2.2442837 |
| 227868_at | 154761 | LOC154761 | 2.6862133 | -1.016597 | 2.1748219 |
| 202023_at | 1942 | EFNA1 | 2.672577 | -1.134444 | 2.140025 |
| 218149_s_at,221123_x_at,222536_s_at,223216_x_at,232693_s_at,23269 | 55893 | FBXO16///ZNF395 | 2.6613812 | -1.0005155 | 2.4505234 |
| 227481_at,242394_at | 154043 | CNKSR3 | 2.6547613 | -1.1260433 | 2.0106716 |
| 221011_s_at | 81606 | LBH | 2.6483796 | -1.0430537 | 1.8323219 |
| 218863_s_at,218864_at,221246_x_at,221747_at,221748_s_at,232750_at | 7145 | TNS1 | 2.6409469 | 1.0196899 | 2.4974587 |
| 209946_at | 7424 | VEGFC | 2.6380374 | -1.1224189 | 2.2622871 |
| 208510_s_at | 5468 | PPARG | 2.632619 | -1.0869524 | 1.3990397 |
| 208891_at,208892_s_at,208893_s_at | 1848 | DUSP6 | 2.6288686 | -1.0732999 | 3.0172298 |
| 223672_at | 84251 | SGIP1 | 2.6123674 | -1.0434889 | 2.89288 |
| 208461_at,230218_at | 3090 | HIC1 | 2.6072962 | 1.0544306 | 1.8571677 |
| 206633_at,211039_at | 1134 | CHRNA1 | 2.6072748 | -1.0815752 | 2.1196766 |

| | | | | | |
|---|---|---|---|---|---|
| 203835_at | 2615 | LRRC32 | 2.5887058 | -1.0504373 | 2.418337 |
| 202855_s_at,202856_s_at,213522_s_at,217685_at,217691_x_at | 9123 | SLC16A3 | 2.575064 | -1.0763892 | 2.6620975 |
| 206084_at,210675_s_at | 5801 | PTPRR | 2.5745492 | 1.1081775 | 1.734538 |
| 228564_at | 375295 | LOC375295 | 2.5697331 | 1.0214471 | 2.6363823 |
| 213280_at | 23108 | GARNL4 | 2.5377605 | 1.4615843 | 1.9120365 |
| 209821_at | 90865 | IL33 | 2.527802 | 1.0623894 | 1.6323873 |
| 206211_at | 6401 | SELE | 2.525342 | 1.4597855 | 1.807039 |
| 205158_at,213397_x_at | 6038 | RNASE4 | 2.5219579 | 1.0859808 | 2.4892704 |
| 208214_at,229277_at,229309_at | 153 | ADRB1 | 2.5138063 | 1.1400461 | 2.536407 |
| 227307_at,228819_at,1561449_at | 90139 | TSPAN18 | 2.5015595 | -1.2474855 | 1.7461693 |
| 209493_at,233025_at,233026_s_at | 23037 | PDZD2 | 2.4981704 | 1.1128467 | 1.9936345 |
| 203417_at | 4237 | MFAP2 | 2.4730103 | -1.105154 | 2.224367 |
| 225283_at | 91947 | ARRDC4 | 2.4702792 | 1.5583578 | 2.5006428 |
| 223315_at | 59277 | NTN4 | 2.4509566 | 1.0857127 | 3.1736004 |
| 219319_at,222123_s_at,222124_at,232669_at,233517_s_at,1555318_at,1 | 64344 | HIF3A | 2.4327266 | 1.0901693 | 2.4273324 |
| 220108_at,243580_at | 9630 | GNA14 | 2.4255335 | -1.0085351 | 1.6824194 |
| 213996_at,228296_at,228788_at | 29799 | YPEL1 | 2.4243898 | 1.0182933 | 2.1329432 |
| 236915_at,237585_at | 441054 | C4orf47 | 2.4220521 | 1.4357066 | 2.383356 |
| 218162_at | 56944 | OLFML3 | 2.411359 | 1.0546969 | 1.9682903 |
| 235953_at | 162963 | ZNF610 | 2.4097505 | 1.0694715 | 2.0069046 |
| 1553635_s_at | 200132 | TCTEX1D1 | 2.391177 | -1.0019665 | 2.0252175 |
| 220092_s_at,220093_at,224694_at,227660_at,234430_at,234832_at | 84168 | ANTXR1 | 2.3854177 | 1.0140164 | 2.203451 |
| 202972_s_at,202973_x_at,217047_s_at,1569024_at,1569025_s_at | 10144 | FAM13A | 2.3677855 | 1.0863153 | 2.238961 |
| 203859_s_at | 5064 | PALM | 2.3641858 | 1.0920115 | 2.0960515 |
| 205846_at,217177_s_at,230250_at,1560105_at | 5787 | PTPRB | 2.359804 | 1.0544599 | 2.5497692 |
| 204135_at,1554965_at,1554966_a_at | 11259 | FILIP1L | 2.3380134 | -1.0937941 | 2.0470176 |
| 219862_s_at | 26502 | NARF | 2.33565 | -1.0587037 | 2.1874118 |
| 205554_s_at,1561336_at | 1776 | DNASE1L3 | 2.3310173 | 1.206035 | 1.8233258 |
| 209774_x_at,230101_at,1569203_at | 2920 | CXCL2 | 2.3308785 | -1.3441355 | 1.7987003 |
| 227263_at,236296_x_at | 541565 | C8orf58 | 2.3261378 | 1.0424777 | 1.8719829 |
| 207196_s_at,243423_at | 10318 | TNIP1 | 2.3091512 | -1.1153599 | 2.2248285 |

| | | | | | |
|---|---|---|---|---|---|
| 206246_at,228499_at | 5210 | PFKFB4 | 2.3066256 | -1.2963716 | 2.205529 |
| 243337_at | 166752 | FREM3 | 2.2856097 | -1.133587 | 1.1894583 |
| 202643_s_at,202644_s_at | 7128 | TNFAIP3 | 2.274235 | 1.1366693 | 2.1153088 |
| 228427_at,232693_s_at | 157574 | FBXO16///ZNF395 | 2.268759 | -1.1465316 | 2.149984 |
| 217867_x_at,222446_s_at | 25825 | BACE2 | 2.2680182 | 1.0146326 | 1.819841 |
| 238429_at | 137835 | TMEM71 | 2.2621398 | 1.2234018 | 2.264053 |
| 222662_at,1552669_at,1552670_a_at | 79660 | PPP1R3B | 2.2617211 | 1.0282257 | 2.077569 |
| 203325_s_at,212488_at,212489_at | 1289 | COL5A1 | 2.2615478 | 1.0658569 | 2.1075532 |
| 205974_at,205975_s_at | 3231 | HOXD1 | 2.2598553 | 1.0077931 | 1.9462303 |
| 203592_s_at | 10272 | FSTL3 | 2.2553558 | 1.0659888 | 1.6256529 |
| 211518_s_at | 652 | BMP4 | 2.24237 | -1.0056616 | 2.0314214 |
| 221221_s_at,1555110_a_at | 26249 | KLHL3 | 2.2348135 | 1.1348771 | 2.271526 |
| 227151_at | 257364 | SNX33 | 2.2322192 | 1.1520879 | 1.8624104 |
| 213349_at,213351_s_at,213352_at,227112_at | 23023 | TMCC1 | 2.207695 | 1.0208389 | 2.026031 |
| 235548_at | 164284 | APCDD1L | 2.1963515 | 1.1245165 | 2.0158021 |
| 1555366_at | 100131275 | NSAP11 | 2.1921139 | -1.1967434 | 1.8107357 |
| 220942_x_at,221533_at,223193_x_at,224345_x_at | 26355 | FAM162A | 2.1745884 | 1.0496544 | 2.215231 |
| 225803_at,241762_at,241763_s_at | 114907 | FBXO32 | 2.1691191 | -1.2190872 | 1.7193284 |
| 219165_at,232302_at | 64236 | PDLIM2 | 2.1648476 | -1.0689782 | 1.9526 |
| 219049_at | 55790 | CSGALNACT1 | 2.162727 | -1.0311981 | 1.610716 |
| 216598_s_at | 6347 | CCL2 | 2.1600096 | -1.0595535 | 1.8255678 |
| 204288_s_at,220858_at,225728_at,233720_at,237285_at,1555472_at,155 | 8470 | SORBS2 | 2.1583967 | -1.0879725 | 1.890009 |
| 223423_at | 26996 | GPR160 | 2.1523507 | 1.4074777 | 1.511825 |
| 204906_at,212912_at,1557970_s_at | 6196 | RPS6KA2 | 2.149721 | -1.0137899 | 1.818362 |
| 207547_s_at,209074_s_at | 11170 | FAM107A | 2.1425042 | 1.0596012 | 2.0806036 |
| 209908_s_at,209909_s_at,220406_at,220407_s_at,228121_at | 7042 | TGFB2 | 2.131523 | -1.0832423 | 2.0179913 |
| 221497_x_at,223045_at,223046_at,224314_s_at | 54583 | EGLN1 | 2.1310742 | -1.0906855 | 1.9458026 |
| 203758_at | 1519 | CTSO | 2.1273916 | -1.0197392 | 1.7217008 |
| 207174_at | 2262 | GPC5 | 2.1257377 | -1.1293306 | 1.6161052 |
| 201032_at | 10904 | BLCAP | 2.1253934 | -1.0865993 | 1.8260674 |
| 205141_at | 283 | ANG | 2.1212332 | 1.3270131 | 2.0240164 |

| | | | | | |
|---|---|---|---|---|---|
| 225955_at | 284207 | METRNL///LOC653506 | 2.1209793 | -1.1133599 | 1.5940164 |
| 225955_at | 653506 | METRNL///LOC653506 | 2.1209793 | -1.1133599 | 1.5940164 |
| 224602_at,224604_at,224605_at | 401152 | C4orf3 | 2.1202826 | -1.0400119 | 2.299671 |
| 229872_s_at | 100132999 | LOC100132999 | 2.119753 | 1.0120751 | 1.5495505 |
| 203504_s_at,203505_at,216066_at,1570279_at | 19 | ABCA1 | 2.1100938 | 1.2356447 | 2.3568194 |
| 206750_at,226206_at | 7975 | MAFK | 2.1041422 | -1.032114 | 1.897115 |
| 220765_s_at | 55679 | LIMS2 | 2.1037471 | 1.0523642 | 1.7651325 |
| 232090_at,235891_at | 100128178 | LOC100128178 | 2.1018226 | -1.0440995 | 2.1785946 |
| 206039_at | 9363 | RAB33A | 2.1001346 | -1.0072429 | 1.5192027 |
| 202403_s_at,202404_s_at,229218_at | 1278 | COL1A2 | 2.0951462 | -1.1576678 | 1.9340343 |
| 230800_at | 196883 | ADCY4 | 2.0943985 | -1.0339007 | 1.875875 |
| 225956_at,225957_at,238476_at,1554229_at | 153222 | C5orf41 | 2.0924127 | 1.0739126 | 1.9774675 |
| 202331_at | 593 | BCKDHA | 2.086277 | 1.0938169 | 1.8832657 |
| 227285_at | 148523 | C1orf51 | 2.085083 | -1.1474195 | 2.6896985 |
| 221016_s_at | 83439 | TCF7L1 | 2.0803952 | 1.0203443 | 1.6747903 |
| 203438_at,203439_s_at | 8614 | STC2 | 2.0640998 | 1.293725 | 2.1740055 |
| 218723_s_at,228193_s_at,239827_at | 28984 | C13orf15 | 2.061145 | -1.0670787 | 1.6759852 |
| 220753_s_at | 51084 | CRYL1 | 2.0590684 | 1.0620204 | 2.0383368 |
| 209034_at | 10957 | PNRC1 | 2.0540316 | 1.0597006 | 1.8538456 |
| 203824_at | 7103 | TSPAN8 | 2.050393 | 1.3287568 | 2.1017601 |
| 212358_at,235243_at | 25999 | CLIP3 | 2.0453627 | 1.0572242 | 1.8384871 |
| 224647_at,224649_x_at,224651_at,224652_at,1554694_at | 219771 | CCNY | 2.0426536 | -1.048578 | 1.8834214 |
| 201473_at | 3726 | JUNB | 2.041329 | 1.0165668 | 1.8109099 |
| 202149_at,202150_s_at,1569020_at | 4739 | NEDD9 | 2.0344055 | -1.0132056 | 2.1341426 |
| 225898_at | 84058 | WDR54 | 2.032354 | 1.0555876 | 2.0671153 |
| 238865_at | 132430 | PABPC4L | 2.0269117 | 1.1380436 | 1.8523573 |
| 231868_at | 57594 | HOMEZ | 2.0247905 | 1.0693351 | 1.5284432 |
| 221211_s_at | 56911 | C21orf7 | 2.0194871 | 1.0919338 | 1.7180967 |
| 220111_s_at,234718_at | 57101 | ANO2 | 2.0129318 | 1.2785292 | 2.040816 |
| 229523_at,238128_at | 645369 | TTMA | 2.011496 | 1.1876391 | 1.6046479 |
| 214719_at | 283537 | SLC46A3 | 2.0113132 | -1.3414589 | 1.1275975 |

| | | | | | |
|---|---|---|---|---|---|
| 205150_s_at,205151_s_at | 9865 | KIAA0644 | 2.0057852 | 1.2285626 | 1.486554 |
| 212154_at,212157_at,212158_at | 6383 | SDC2 | 2.005355 | -1.0155405 | 1.6271296 |

**Supplemental Table S3A. Primers list for expression analysis**

| | |
|---|---|
| ANGPTL4fw | GCAGGATCCAGCAACTCTTC |
| ANGPTL4rv | AAACTGGCTTTGCAGATGCT |
| HIF1αfw | CGAGATGCAGCCAGATCTCGGCGAAGTAA |
| HIF1αrv | CTCTCATTTCCTCATGGTCACATGGATG |
| PPARβ/δfw | TCATTGCGGCCATCATTCTGTGTG |
| PPARβ/δrv | TTCGGTCTTCTTGATCCGCTGCAT |
| PDK4fw | TGCAATTGGTTAAAAGCTGGT |
| PDK4rv | CATCTGGGCTTTTCTCATGG |
| CPT1Afw | ACAGTCGGTGAGGCCTCTTATGAA |
| CPT1Arv | CTTGCTGCCTGAATGTGAGTTGG |
| SLC25A20fw | GTTATCTGGCGTATTCACCACA |
| SLC25A20rv | GTCCAGTGTACTTGCTTTCTCC |
| VEGFAfw | GCGGAGAAAGCATTTGTTTGT |
| VEGFArv | CGGCTTGTCACATCTGCAAC |
| SLC2A1fw | GGTTGTGCCATACTCATGACC |
| SLC2A1rv | CACATAGGACATCCAGGGTAGC |
| ADSSL1fw | CAGGCAGGAAGAGGCGCTGC |
| ADSSL1rw | GGCCAGCGCAGTGAATCCGT |
| ANGPTL4fw (1$^{st}$ exon) | GCCTCAGCGGATGGAGATTT |
| ANGPTL4rv (1$^{st}$ intron) | GAGTTGCTGGATCCTGCTGT |
| ANGPTL4fw (2$^{nd}$ intron) | GAGGTGGTGAGAACTGGACG |
| ANGPTL4rv (3$^{rd}$ exon) | GGTCTAGGTGCTTGTGGTCC |
| ANGPTL4fw (5$^{th}$ intron) | TTCTAGTGGGGACAGAGGCA |
| ANGPTL4rv (6$^{th}$ exon) | CCCGTGATGCTATGCACCTT |

**Supplemental Table S3B. Primers for site-directed mutagenesis of the HRE and PPRE**

| | |
|---|---|
| mutHRE1 | 3’ - GCACACCCAAGGGCTAGATCTCAGGCTGCCACTCAT |
| mutHRE2 | 5’ - CCGAGTAGCTGTAAGAGATCTCCACCACACCCGGCT |
| mutHRE3 | 5’ - GGGGGAAAGAAGAGTCAGATCTCCCACGGTTCGTAG |
| mutPPRE1 | 5’ - CAGGCCCGCCAAGTAGAGATCTGTTCAGAGCTGGGA |
| mutPPRE2 | 5’ - GATGGGAGGAAAGTAGAGATCTGGGGAGATGCCTGA |
| mutPPRE3 | 3’- CCAGCCGGAAAAGTAGAGATCTGGTCGAAATGAGTC |

**Supplemental Table S3C. Primers list for ChIP assay**

| | |
|---|---|
| ANGPTL4 PPRE at 3rd intron +3500fw | CCTTACTGGATGGGAGGAAAG |
| ANGPTL4 PPRE at 3rd intron +3500rv | CCCAGAGTGACCAGGAAGAC |
| ANGPTL4 Negative Control for PPARβ/δ +15000fw | GCCTATAGCCTGCAGCTCAC |
| ANGPTL4 Negative Control for PPARβ/δ +15000rv | CAAGTGGAGAAGGGTACGGA |
| ANGPTL4 HIF1α binding site for H3K27ac -1800fw | TAGGGGAATGGGTAGGGAAG |
| ANGPTL4 HIF1α binding site for H3K27ac -1800rv | AGTTCTCAGGCAGGTGGAGA |
| ANGPTL4 PPARβ/δ binding site for H3K27ac +3200fw | TAGCATCTCAGCGTGGTCAG |
| ANGPTL4 PPARβ/δ binding site for H3K27ac +3200rv | TGAGCCAAGAGTCTGGGAGT |
| ANGPTL4 TSS for Pol II fw | ACCGACCTCCCGTTAGCCCC |
| ANGPTL4 TSS for Pol II rv | CGGAGAACCAGCCCTGGGGA |

**Supplemental Table S3D. Primers and TaqMan probe for 3C assay**

| Target analysis | |
|---|---|
| ANGPTL4 Target Anchor Fw (TSS-3kb, HRE) | TGGTTTTCTGTGCCAGCCTC |
| ANGPTL4 Target Anchor TaqMan probe | (6-FAM) CCTTCCTGACAGGGTATAG (MGB) |
| ANGPTL4 Control Anchor Fw1 (TSS-4kb) | AACACGGCAAAAATCCTGTC |
| ANGPTL4 Control Anchor TaqMan probe1 | (6-FAM) TGCCTATAGTCCCAGCTGCT (MGB) |
| ANGPTL4 Control Anchor Fw2 (TSS+6kb) | CCAGAGAGGCTTTGCAGATT |
| ANGPTL4 Control Anchor TaqMan probe2 | (6-FAM) CCTACTCCCTCCCACACTCA (MGB) |
| ANGPTL4 Rv (TSS+5.3kb, PPRE) | CCTGACCACGCTGAGATGCTA |
| ANGPTL4 Rv (TSS-10kb) | AATGGCTCACGCCTGTAATC |
| ANGPTL4 Rv (TSS-8kb) | TCGGTCCCTGAAAGGAGATA |
| ANGPTL4 Rv (TSS-6kb) | CCCGTACTCCCACACGTACT |
| ANGPTL4 Rv (TSS+1kb) | CCAGGCAAGGGAGTCTTTCT |
| ANGPTL4 Rv (TSS+4kb) | TGTAGAGACGGGGGTTTCAC |
| ANGPTL4 Rv (TSS+15kb) | GCCTCCCTCCCTCTATCATC |
